# Transcriptome, proteome and draft genome of *Euglena gracilis*

**DOI:** 10.1186/s12915-019-0626-8

**Published:** 2019-02-07

**Authors:** ThankGod E. Ebenezer, Martin Zoltner, Alana Burrell, Anna Nenarokova, Anna M. G. Novák Vanclová, Binod Prasad, Petr Soukal, Carlos Santana-Molina, Ellis O’Neill, Nerissa N. Nankissoor, Nithya Vadakedath, Viktor Daiker, Samson Obado, Sara Silva-Pereira, Andrew P. Jackson, Damien P. Devos, Julius Lukeš, Michael Lebert, Sue Vaughan, Vladimίr Hampl, Mark Carrington, Michael L. Ginger, Joel B. Dacks, Steven Kelly, Mark C. Field

**Affiliations:** 1 School of Life Sciences, University of Dundee, Dundee, DD1 5EH UK; 2 Department of Biochemistry, University of Cambridge, Cambridge, CB2 1QW UK; 3 Department of Biological and Medical Sciences, Faculty of Health and Life Sciences, Oxford Brookes University, Oxford, OX3 0BP UK; 4 Biology Centre, Institute of Parasitology, Czech Academy of Sciences, and Faculty of Sciences, University of South Bohemia, 37005 České Budějovice, Czech Republic; 5 Department of Parasitology, Faculty of Science,, Charles University, BIOCEV, 252 50 Vestec, Czech Republic; 6 Cell Biology Division, Department of Biology, University of Erlangen-Nuremberg, 91058 Erlangen, Germany; 7 Centro Andaluz de Biología del Desarrollo (CABD)-CSIC, Pablo de Olavide University, Seville, Spain; 8 Department of Plant Sciences, University of Oxford, Oxford, OX1 3RB UK; 9 Division of Infectious Disease, Department of Medicine, University of Alberta, Edmonton, Alberta T6G Canada; 10 Laboratory of Cellular and Structural Biology, The Rockefeller University, New York, NY 10065 USA; 11 Department of Infection Biology, Institute of Infection and Global Health, University of Liverpool, Liverpool, UK; 12 Department of Biological and Geographical Sciences, School of Applied Sciences, University of Huddersfield, Queensgate, Huddersfield, HD1 3DH UK; 13 Department of Life Sciences, The Natural History Museum, Cromwell Road, London, SW7 5BD UK

**Keywords:** *Euglena gracilis*, Transcriptome, Cellular evolution, Plastid, Horizontal gene transfer, Gene architecture, Splicing, Secondary endosymbiosis, Excavata

## Abstract

**Background:**

Photosynthetic euglenids are major contributors to fresh water ecosystems. *Euglena gracilis* in particular has noted metabolic flexibility, reflected by an ability to thrive in a range of harsh environments. *E. gracilis* has been a popular model organism and of considerable biotechnological interest, but the absence of a gene catalogue has hampered both basic research and translational efforts.

**Results:**

We report a detailed transcriptome and partial genome for *E. gracilis* Z1. The nuclear genome is estimated to be around 500 Mb in size, and the transcriptome encodes over 36,000 proteins and the genome possesses less than 1% coding sequence. Annotation of coding sequences indicates a highly sophisticated endomembrane system, RNA processing mechanisms and nuclear genome contributions from several photosynthetic lineages. Multiple gene families, including likely signal transduction components, have been massively expanded. Alterations in protein abundance are controlled post-transcriptionally between light and dark conditions, surprisingly similar to trypanosomatids.

**Conclusions:**

Our data provide evidence that a range of photosynthetic eukaryotes contributed to the *Euglena* nuclear genome, evidence in support of the ‘shopping bag’ hypothesis for plastid acquisition. We also suggest that euglenids possess unique regulatory mechanisms for achieving extreme adaptability, through mechanisms of paralog expansion and gene acquisition.

**Electronic supplementary material:**

The online version of this article (10.1186/s12915-019-0626-8) contains supplementary material, which is available to authorized users.

## Introduction

*Euglena gracilis*, a photosynthetic flagellate, was first described by van Leeuwenhoek in 1684 [1]. There are over 250 known species in the genus *Euglena*, with around 20 predominantly cosmopolitan, including *E. gracilis* [2–5]. *Euglena* spp. are facultative mixotrophs in aquatic environments [6] and many possess a green secondary plastid derived by endosymbiosis of a chlorophyte algae [7]. Amongst the many unusual features of euglenids are a proteinaceous cell surface pellicle [8] and an eyespot [9–14]. Euglenids, together with kinetoplastids, diplonemids and symbiotids, form the Euglenozoa subgroup of the Discoba phylum [15]. Kinetoplastids are best known for the *Trypanosoma* and *Leishmania* lineages [15], important unicellular parasites, while diplonemids have been little studied, yet represent one of the most abundant and diverse eukaryotic lineages in the oceans [16].

*E. gracilis* is thus of importance due to evolutionary history, divergent cellular architecture, complex metabolism and biology, together with considerable potential for biotechnological exploitation [17]. However, the full complexity of euglenid biology remains to be revealed, and the absence of a complete genome sequence or annotated transcriptome has greatly hampered efforts to study *E. gracilis* or to develop genetic tools [17, 18]. Two transcriptomes have been published, one derived from cells grown in light and dark conditions plus rich versus minimal media [17] and a second examining the impact of anaerobic conditions on gene expression [19]. For the most part, these studies focused on the biosynthetic properties of *E. gracilis* and not cellular systems or aspects of protein family evolution. Most recently, a study of low molecular weight RNA populations identified over 200 snoRNAs [20].

Comparisons between euglenozoans such as the free-living bodonids, early-branching trypanosomatids (*Paratrypanosoma confusum*), and parasitic forms have uncovered many genetic changes associated with parasitism [21–24]. Both the cell surface and flagellum of euglenoids are of significant importance to life cycle development, interaction with the environment and, for parasitic trypanosomes, pathogenesis and immune evasion [25, 26]. The surface macromolecules of trypanosomatids are highly lineage-specific with roles in life cycle progression [23, 27–31], but it remains to be determined to what extent *E. gracilis* shares surface proteins or other aspects of biology with the trypanosomatids or how cellular features diverge. Such information is invaluable for determining how parasitism arose in the kinetoplastids.

*E. gracilis* produces a wide range of secondary metabolites, and many of which are of potential commercial value [17]. Furthermore, *E. gracilis* is of considerable promise for biofuel production [32–34], and extremely resistant to conditions such as low pH and high metal ion concentrations, fueling interest as possible sentinel species or bioremediation agents [19, 35–37]. In parts of Asia, *E. gracilis* is cultivated as an important food supplement [38].

*E. gracilis* possesses a complex genome, with nuclear, plastid and mitochondrial components, an overall architecture known for decades. The coding potential of the mitochondrial genome is surprisingly small [39, 40], while the plastid is of more conventional structure [41]. The plastid is the result of a secondary endosymbiotic event, which is likely one of several such events occurring across eukaryotes [42]. Uncertainties concerning the origins of the plastid have remained, and not least of which has been the presence of genes from both red and green algae in the *E. gracilis* nuclear genome [19, 43]. Such a promiscuous origin for photosynthetic genes is not restricted to the euglenids and has been proposed as a general mechanism, colloquially the ‘shopping bag’ hypothesis, whereby multiple endosymbiotic events are proposed and responsible for the range of genes remaining in the nuclear genome, providing a record of such events and collecting of genes, but where earlier symbionts have been completely lost from the modern host [44].

The *E. gracilis* nuclear genome size has been estimated as in the gigabyte range [45–48] and organization and intron/exon boundaries of very few genes described [49–54]. In the kinetoplastids, unusual transcriptional mechanisms, involving the use of *trans*-splicing as a near universal mechanism for maturation of protein-coding transcripts and polycistronic transcription units, have been well described. As *E. gracilis* supports multiple splicing pathways, including conventional and non-conventional *cis*- [52, 53] and *trans-*splicing [55], there is scope for highly complex mechanisms for controlling expression, transcription and mRNA maturation [56], but how these are related to kinetoplastids is unclear.

We undertook a polyomic analysis of the Z1 strain of *E. gracilis* to provide a platform for improved understanding of the evolution and functional capabilities of euglenids. Using a combination of genome sequencing, together with pre-existing [17] and new RNA-seq analysis, proteomics and expert annotation, we provide an improved view of *E. gracilis* coding potential and gene expression for greater understanding of the biology of this organism.

## Results and discussion

### Genome sequencing of *Euglena gracilis*

We initiated sequencing of the *E. gracilis* genome using Roche 454 technology. The early assemblies from these data indicated a large genome in excess of 250 Mb and that data coverage was low. We turned to the Illumina platform and generated data from multiple-sized libraries, as well as a full lane of 150 bp paired-end sequences. These data were assembled as described in methods and as previously [48] and latterly supplemented with PacBio data generously donated by colleagues (Purificatión Lopéz-García, David Moreira and Peter Myler, with thanks). The PacBio data however failed to improve the assembly quality significantly, presumably due to low coverage.

Our final draft genome assembly has 2,066,288 sequences with N_50_ of 955 (Table 1), indicating significant fragmentation. The estimated size of the single-copy proportion of the genome is 140–160 mb and the estimated size of the whole haploid genome is 332–500 mb. This is consistent with several estimates from earlier work (e.g. [57]), *albeit* based here on molecular sequence data rather than estimates of total DNA content. Using the core eukaryotic genes mapping approach (CEGMA) [58], we estimate that the genome assembly, or at least the coding sequence proportion, is ~ 20% complete. Hence, this assembly could only support an initial analysis of genome structure and is unable to provide a full or near full open reading frame catalog (Table 2). The heterozygosity, size and frequency of low complexity sequence hampered our ability to assemble this dataset (see the “Materials and Methods” section for more details). The size and frequency of low-complexity sequence clearly precluded assembly of our dataset from Illumina reads, and significantly, PacBio data had no significant impact on assembly quality. Due to the large proportion of low-complexity sequence, any estimate for the size of the genome is very much an approximation.

**Table 1:** Statistics of genome assembly

Parameter	
Number of sequences	2,066,288
Median sequence length	457
Mean sequence length	694
Max sequence length	166,587
Min sequence length	106
No. sequence > 1kbp	373,610
No. sequence > 10kbp	1459
No. sequence > 100kbp	2
No. gaps	0
Bases in gaps	0
N50	955
Combined sequence length	1,435,499,417

Following the assembly process, over two million sequences were retained, with a median sequence length of 457 bp

**Table 2:** CEGMA analysis of selected datasets

Assembly	Organism	Gene status	Prots	%Completeness	Total	Average	%Ortho
Genome	*E. gracilis*	Complete	22	8.87	37	1.68	54.55
		Partial	50	20.16	89	1.78	56
	*T. brucei*	Complete	196	79.03	259	1.32	24.49
		Partial	205	82.66	282	1.38	28.29
	*L. major*	Complete	194	78.23	220	1.13	11.34
		Partial	204	82.26	245	1.2	15.69
Transcriptome	*E. gracilis*	Complete	187	75.4	390	2.09	65.78
		Partial	218	87.9	506	2.32	69.72
	*T. brucei*	Complete	190	76.61	393	2.07	60
		Partial	205	82.66	448	2.19	63.41
	*L. major*	Complete	133	53.63	275	2.07	64.66
		Partial	194	78.23	405	2.1	64.43

Comparisons for CEGMA scores between *E. gracilis, T. brucei* and *L. major* as an estimate of ‘completeness’ based on 248 CEGs. *Prots* number of 248 ultra-conserved CEGs present in genome, *%Completeness* percentage of 248 ultra-conserved CEGs present, *Total* total number of CEGs present including putative orthologs, *Average* average number of orthologs per CEG, *%Ortho* percentage of detected CEGs that have more than 1 ortholog, *Complete* those predicted proteins in the set of 248 CEGs that when aligned to the HMM for the KOG for that protein family, give an alignment length that is 70% of the protein length. i.e. if CEGMA produces a 100 amino acid protein, and the alignment length to the HMM to which that protein should belong is 110, then we would say that the protein is “complete” (91% aligned), *Partial* those predicted proteins in the 248 sets that are incomplete, but still exceeds a pre-computed minimum alignment score. Keys are as described [58]

**Fig. 1:**
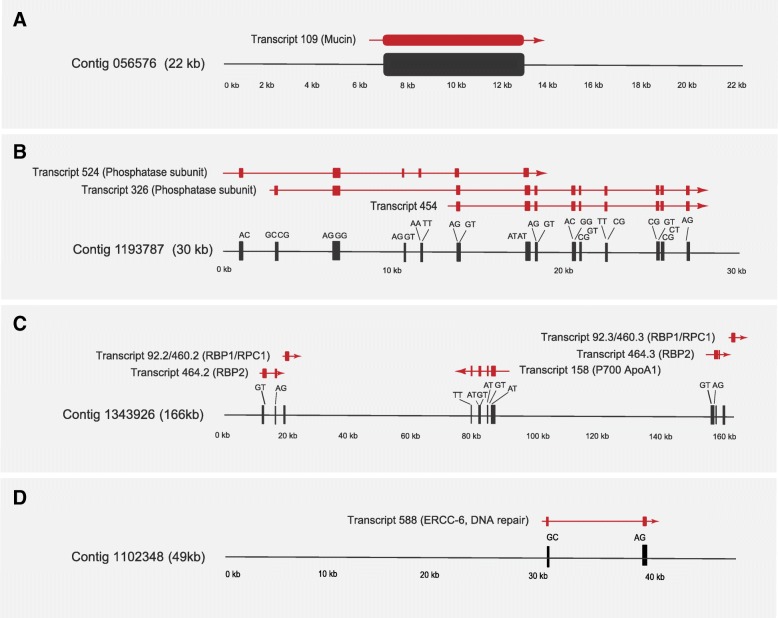
**a**–**d** *Euglena gracilis* exon structure. The predicted gene structure of several selected contigs is shown, including the mapped transcripts (red), predicted splice sites and intergenic regions. Note that transcripts 524 and 326, (panel **b**) which encompass essentially the same portions of the genome, demonstrate possible differential exon inclusion, indicating differential open reading frame organisation and possible alternate splicing. Black boxes indicate exons, with predicted splice site dinucleotides indicated above. Transcripts are shown as arrows with the arrowhead indicating the predicted direction of transcription. Protein product annotations are indicated in parentheses. Contig sizes are shown in kilobase; note that each contig is not drawn to the same scale. Further examples of predicted contig gene organisation are given in Additional file 1: Figure S1

Restricting analysis to contigs > 10 kb, where some features of overall gene architecture could be inferred, we identified several unusual aspects of genome structure (Table 3, Fig. 1, Additional file 1: Figure S1). These contigs encompassed about 22 Mb of sequence, but with only 135 genes predicted based on Exonerate [59], this suggests an extremely low gene density of < 1%, similar to that in *Homo sapiens*. In those contigs that possess predicted coding sequence, there was frequently more than one open reading frame (ORF), suggesting gene clusters present within large expanses of non-coding sequence (e.g. Contig11343926, Fig. 1c), but with the caveat that we have sampled a very small proportion of total ORFs (Table 3). It is also possible that some genes were not predicted due to absence of expression under the conditions we used for RNA-seq, though we consider this likely a minor contribution as multiple culturing conditions were included within the final RNA-seq dataset (see below). Most identified genes are predicted to be *cis*-spliced and most introns are conventional, with a smaller proportion of intermediate and non-conventional splice sites (consistent with [57]). Some introns appear very large compared to the coding sequence contained between them (Contig 1102348, Transcript 588, Fig. 1d). Furthermore, some genes are apparently unspliced (Fig. 1a; Contig 056576, Transcript 109) and there is evidence for alternate splicing (Fig. 1b; Contig 1193787, Transcripts 326, 454 and 524). Evidence for alternate spicing was described earlier [19], but it was based on RNA-seq data without a genomic context, unlike here. The near complete absence of *cis*-splicing from bodonids and trypanosomatids clearly reflects loss post-speciation of these lineages from euglenids and removed a considerable mechanism for generation of proteome diversity [60]. The biological basis for the extreme genome streamlining in the trypanosomatids versus *Euglena* is unclear.

**Table 3:** Characteristics of contigs assembled with length exceeding 10 kb

Contigs	Total contigs analysed > 10 kb	1459
	Total nucs in contigs analysed	22 Mb
	Contigs with CDS	53
	Percent contigs with CDS	3.6
CDS	Number analysed	135
	Average length	3790
	Total length	481,369
Exons	Number of exons analysed	421
	Average Length	174.54
	Median Length	112
	Total Length	73,482
	Average per predicted CDS	3.85
Introns	Total introns analysed	271
	Average length	1027.14
	Median length	598
	Total length	278,354
	Introns per predicted CDS	2.01
	Number/percent conventional	218/80.1
	Number/percent intermediate	30/11.1
	Number/percent non-conventional	23/8.5
	Percent nucleotides in CDS (exon)	0

The contigs were ranked by size and those exceeding 10 kbp extracted and analyzed for length, coding sequence, exon structure and other features

We also sequenced and assembled an *E. gracilis* transcriptome using a combination of in-house generated sequence and publicly available data [17]. This strategy had the advantage of focusing on coding sequence, as well as including data from multiple environmental conditions (see [17], which used dark, light conditions and rich or minimal media and data from here that used distinct media and also light and dark conditions), to increase the likelihood of capturing transcripts, and represents a third analysis, *albeit* incorporating raw reads from previous work [17].

Over 32,000 unique coding transcripts were predicted by [17], which compares well with this new assembly and which accounted for 14 Mb of sequence overall. Of these transcripts, approximately 50% were annotatable using UniRef, and over 12,000 were associated with a GO term. In a second report, Yoshida et al. [19], assembled 22 Mb of coding sequence within 26,479 likely unique components, with about 40% having assignable function based on sequence similarity to Swiss-Prot.

The total number of coding sequence nucleotides in our new assembly was > 38 Mb, with a mean length of 869 bases and 36,526 unique coding sequences (Table 4). This is a significant improvement over 391 bases reported by [17], and comparable to [19], albeit with a significant increase in total sequence assembled. Transcriptome coverage of ORFs was, as expected, significantly superior to the genome, and CEGMA indicated 87.9% recovery (the *Trypanosoma* *brucei* genome is 82.66%) (Tables 2 and 4).

**Table 4:** Assembly statistics for the transcriptome

Transcripts		Coding sequence (CDS)		Proteins	
Number of sequences	72,509	Number of sequences	36,526	Number of proteins	36,526
Median sequence length	540	Median sequence length	765	Median protein length	254
Mean sequence length	869	Mean sequence length	1041	Mean protein length	346
Max sequence length	25,763	Max sequence length	25,218	Max protein length	8406
Min sequence length	202	Min sequence length	297	Min protein length	98
No. sequence > 1kbp	19,765	No. sequence > 1kbp	13,991	No. proteins > 1kaa	1290
No. sequence > 10kbp	25	No. sequence > 10kbp	24	N50	471
No. sequence > 100kbp	0	N50	1413		
No. gaps	0	Combined sequence length	38,030,668		
Bases in gaps	0				
N50	1242				
Combined sequence length	63,050,794				

We also compared the completeness of our transcriptome with the two published transcriptomes of *E. gracilis* [17, 19]. We used TransDecoder (v2.0.1) [61] to translate nucleotide transcripts to proteins and then excluded duplicated proteins with CD-HIT utility (v4.6) with standard parameters [62]. The final comparison, made by BUSCO (v2.0.1) [63] with the eukaryotic database, is shown as Additional file 1: Figure S12. Note that all three studies report similar statistics, including concordance in the cohort of BUSCOs not found; these may have failed to be detected or genuinely be absent. Given that 19 BUSCOs were not found in concatenated data (i.e. all three assemblies), with between four to eight missing BUSCOs specific to individual assemblies, it is highly likely that these datasets are robust while also indicating saturation in terms of achieving ‘completeness’, together with possible limitations with BUSCO for divergent species such as *E. gracilis*.

Comparisons between genome and transcriptome assembly sizes confirmed the very small coding component, with genome contigs containing significantly less than 1% coding sequence, despite the total number of *E. gracilis* ORFs (36526) being two to three times greater than *Bodo saltans* (18963), *T. brucei* (9068) or *Naegleria gruberi* (15727) [64–66]. This is in full agreement with earlier estimates of genome versus transcriptome size [17] as well as estimates of the proportion of coding and total genomic sequence discussed above. This is also similar to other large genomes and, specifically, *Homo sapiens*. Blast2GO and InterProScan annotated over 19,000 sequences with GO terms, a proportion similar to previous reports (Additional file 1: Figure S2, [17, 19]).

In addition to the formal analysis and calculation of the numbers of unique sequences, our annotation of the transcriptome adds additional confidence that the dataset is a good resource:

(i) Most expected metabolic pathways could be reconstructed, with very few exceptions,
(ii) Major known differences between kinetoplastids and *Euglena* were identified, supporting sampling to a deep level,
(iii) For most analyzed protein complexes, all subunits or none were identified, indicating that partial coverage of components is likely rare.

Overall, we conclude that the transcriptome is of sufficient quality for robust annotation and prediction and encompasses more than previous datasets.

### Post-transcriptional control of protein expression

Trypanosomatids exploit post-transcriptional mechanisms for control of protein abundance, where essentially all genes are produced from polycistronic transcripts via *trans*-splicing. To improve annotation and investigate gene expression in *E. gracilis*, we conducted comparative proteomic analysis between light and dark-adapted *E. gracilis* but retained in the same media and temperature. Previous work suggested that control of protein abundance may be post-transcriptional [67, 68], but analysis was limited and did not consider the entire proteome, while a separate study identified some changes to mRNA abundance under low oxygen tension [19]. Under these well-controlled conditions, however, significant changes to the proteome were expected. We confirmed by UV/VIS spectroscopy and SDS-PAGE that photosynthetic pigments were lost following dark adaptation and that ensuing ultrastructural changes, i.e. loss of plastid contents, were as expected (Additional file 1: Figure S3). Total protein extracts were separated by SDS-PAGE with 8661 distinct protein groups (representing peptides mapping to distinct predicted ORFs, but which may not distinguish closely related paralogs) identified. Ratios for 4681 protein groups were quantified (Additional file 2: Table S1) including 384 that were observed in only one state (232 in light and 152 in dark). In parallel, we extracted RNA for RNA-seq analysis; comparing transcript hits with protein groups identified 4287 gene products with robust information for both protein and RNA abundance.

**Fig. 2:**
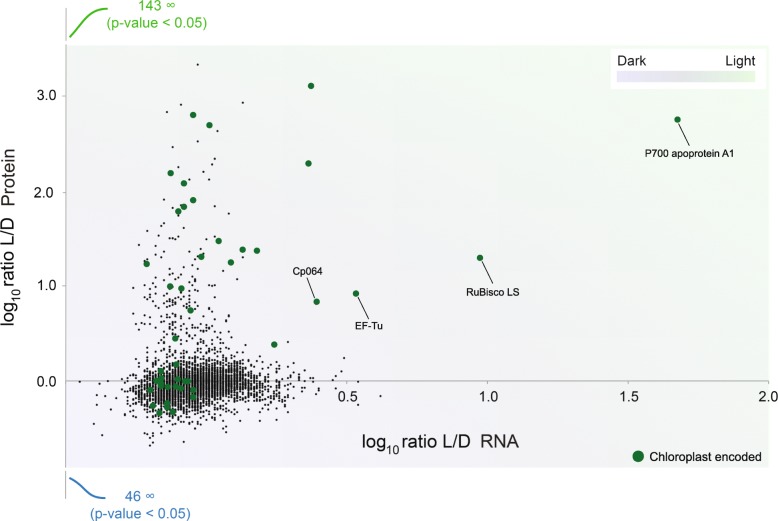
Expression level changes induced by light are mainly post-transcriptional. Alterations to the transcriptome and proteome in response to ambient light or complete darkness were analysed using RNA-seq and SILAC/LCMS^2^ proteomics respectively. Data are plotted for individual transcripts/polypeptides as the log_10_ ratio between the two conditions, light (L) and dark (D), with protein on the *y*-axis and RNA on the *x*-axis. The presence of a number of proteins that were detected exclusively under one or other condition (hence infinite ratio) are indicated in green (for light) and blue (for dark). With the exception of a few transcripts, which are plastid encoded (green dots), there is little alteration to RNA abundance, but considerable changes to protein levels. Raw data for transcriptome/proteome analysis are provided in Additional file 3

Correlations between changes to transcript and protein abundance were remarkably poor (Fig. 2, Additional file 1: Figure S3, Additional file 2: Table S1), consistent with some much smaller earlier studies [67, 68] and broadly with the more extensive study reported in [19]. BLAST analysis revealed that those transcripts where differential abundance did correlate with protein abundance are encoded by the chloroplast genome, including several photosystem I proteins, i.e. P_700_ chlorophyll apoprotein A_1_, the large subunit of ribulose-1,5-bisphosphate carboxylase/oxygenase (RuBisCO) and chloroplast encoded EF-Tu. Nuclear elongation factors are not influenced by switching growth conditions from dark to light [69], consistent with our finding of no differential expression of nuclear EF-1α, while both the chloroplast EF-Tu protein and corresponding transcript (EG_transcript_1495) are highly upregulated by light. This absence of transcriptional control for proteome changes between these two conditions is highly similar to that reported for the kinetoplastids, despite the presence of widespread *cis*-splicing and a sparse genome that likely precludes extensive polycistronic transcription. It remains to be determined if this is a general feature for *E. gracilis* or only for certain environmental cues; a cohort of genes are strongly impacted at the RNA level when comparing aerobic to anaerobic transcripts for example, but in that instance none of these transcripts were plastid-encoded nor was a protein analysis performed [19].

### Ancestry of *Euglena gracilis* genes

**Fig. 3:**
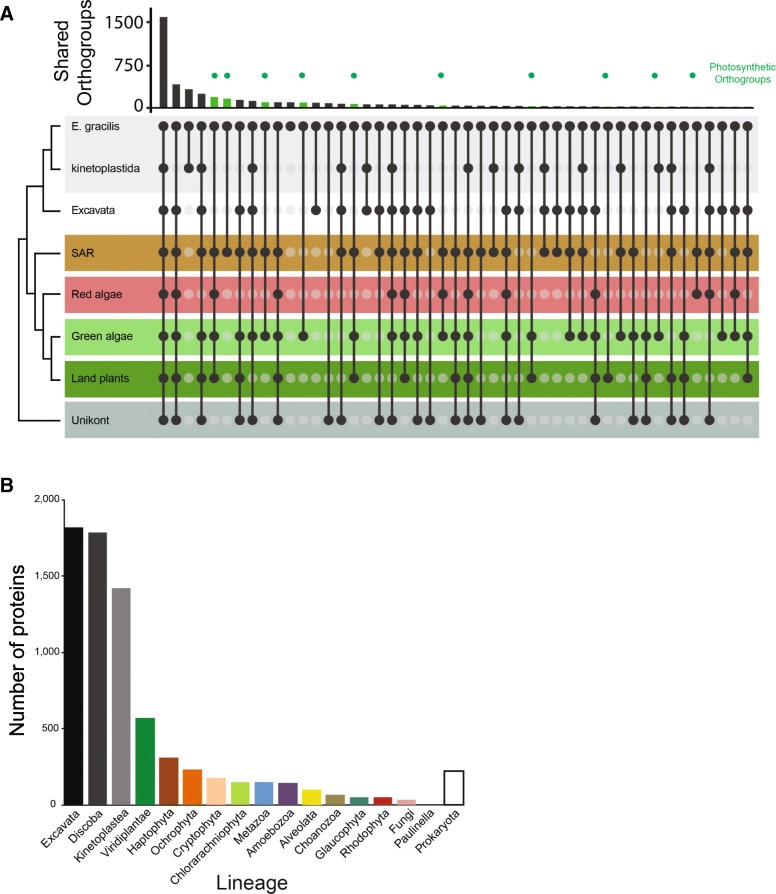
*Euglena gracilis* shares orthologs with a diverse array of lineages. Panel **a** (top): Histogram of *E. gracilis* orthologous groups clustering with selected eukaryotic lineages as determined with OrthoFinder. The *x*-axis shows the number of orthogroups and *y*-axis shows the taxon groupings representative of selected eukaryotic groups. Histogram bars highlighted in green indicate orthogroups shared with photosynthetic organisms. Panel **a** (lower): taxa sharing orthogroups with *E. gracilis*, where black circles correspond to the presence of orthogroup members while light gray circles correspond to the absence of orthogroup members in the genome. Black tie bars linking black circles are for clarity only. Eukaryotic taxon groupings are colored accordingly: gray, *Euglena* and kinetoplastida; white, other members of the Excavata excluding Euglenozoa; brown, SAR, pink, red algae; light green, green algae; dark green, land (vascular) plants and dark gray, Unikona. An expanded version of this figure, broken down by species is given as Additional file 1: Figure S4. Panel **b**: The number of *E. gracilis* proteins that clustered (BS > 75%) in their single-protein phylogenetic tree with taxonomic group are indicated on the x-axis

We used two different approaches to analyze the evolutionary origin of genes predicted from the *E. gracilis* transcriptome. Firstly, we used OrthoFinder [70] to identify *E. gracilis* ortholog gene families shared across eukaryotes and those restricted to specific taxonomic groupings (Fig. 3a, Additional file 1: Figure S4). As expected, the largest proportion was represented by all supergroups and dominated by core metabolic, structural and informational processes, consistent with previous work [19]. A second cohort is shared between *E. gracilis* and other excavates. These classes are broadly within the relative frequencies of previous analyses of excavate genomes [19, 71]. A third cohort represents nuclear transfer of endosymbiotic genes from acquisition of the plastid, and consequently, the genome is a complex mosaic as all eukaryotic genomes also harbour genes driven from the mitochondrial endosymbiont. GO terms associated with orthogroups indicated increased frequency of regulatory function genes in green/secondary plastid orthogroups (Additional file 1: Figure S2). Previous transcriptome studies reported the presence of pan-eukaryotic genes and cohorts shared with kinetoplastids and plants [17, 19], but these were not analyzed in detail, and specifically did not determine which plant taxa were acting as potential gene donors. This is important in terms of understanding the origins of the *Euglena* plastid and where earlier data suggested the presence of a diverse set of genes from at least green, red and brown algae ([43, 72]). Particularly relevant here is that plastid acquisition in euglenoids is relatively recent [73].

To address this question, we employed a second approach, in which we performed exhaustive analysis to establish phylogenetic ancestry of individual proteins from the predicted *Euglena* proteome by generating single-protein phylogenies. Unlike the analyses of orthogroup sharing, this second approach can be used only for a subset of proteins with a sufficiently robust phylogenetic signal, but also allows determination of the gene ancestry; moreover, this is applicable for members of complex gene families. From all predicted *E. gracilis* proteins only 18,108 formed reliable alignment (> 75 positions) with more than two sequences from our custom database, which comprised 207 taxa in total (Additional file 3 Table S2) and was used for tree construction. In 4087 trees, *E. gracilis* formed a robust (bootstrap support > = 75%) sister relationship with a taxonomically homogeneous clade (Fig. 3b). Of these, 1816 (44%) were related to one of the lineages of Excavata and 1420 (35%) were related specifically to kinetoplastids. This major fraction represents mostly the vertically inherited component of the genome. The largest non-vertical component forms a group of 572 (14%) proteins related to green plants and green algae, likely representing genes acquired by endosymbiotic gene transfer from the *Euglena* secondary chloroplast, but it should be noted that the direction of transfer cannot be objectively determined. This category is followed by four groups related to the algal groups: haptophytes, cryptophytes, ochrophytes and chlorarachniophytes. While many proteins within the chlorarachniophyte group may represent mis-assigned genes related to green algae, these relatively large numbers related to the three brown-algal groups (723 in total) suggests that these algae contributed considerably to the *E. gracilis* genome and that the process of chloroplast endosymbiosis was complex (see below). On the other hand, the number of proteins related to red algae and glaucophytes (50 and 53) is near negligible. Proteins in groups shared with prokaryotes (220) and non-photosynthetic eukaryotes, e.g. Metazoa (149) and Amoebozoa (145), are most probably the result of horizontal gene transfers, differential gene losses or artifacts caused by biased phylogenetic reconstructions or contaminations in the data sets used to construct the custom database. The robust nature of our analysis, being restricted to phylogenetically well-resolved trees, provides an additional level of confidence to the concept of multiple origins for LGT genes.

It was initially thought that plastid-possessing organisms would overwhelmingly possess nuclear genes derived by transfer from the endosymbiont corresponding to the plastid currently present, but this has been challenged [74, 75]. While contributions from multiple algal lineages could be explained by incomplete phylogenetic sampling, this is also consistent with the ‘shopping bag’ hypothesis, which proposes an extended process of transient endosymbiosis and gene acquisition by the host prior to the present configuration [44, 75] and which is likely a quite general phenomenon and occurs in many lineages. Our analysis strongly supports the concept of sequential endosymbiotic events.

### Expansive paralog families

**Fig. 4:**
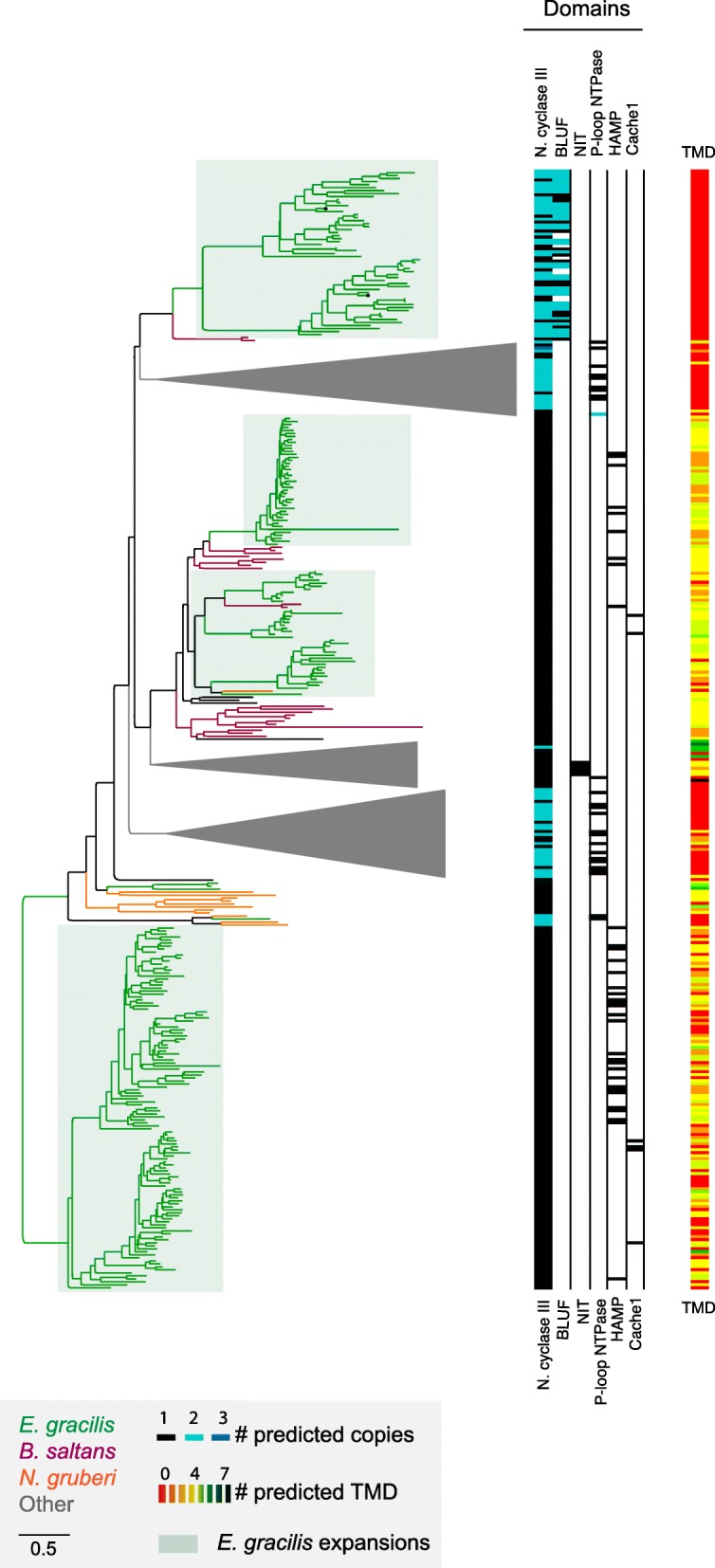
Large paralog gene families are present in the *Euglena gracilis* genome. Several orthogroups contain many *E. gracilis* paralogs. The phylogenetic distribution of one large orthogroup, the nucleotidylcyclase III domain-containing proteins, is shown**.** Lineage groupings are colour coded: gray, all eukaryotes (and collapsed for clarity); red, *N. gruberi*; amber, *B. saltans*; and green, *E. gracilis*. Clades containing only *Euglena* sequences are boxed in green. Each sequence has been assigned a domain composition (colour gradient black to teal to blue), number of predicted trans-membrane domains (colour coded red to orange to black gradient). To obtain this phylogenetic tree, sequences with likely low coverage (less than 30% of the length of the overall alignment) were removed during alignment to avoid conflicting homology or artefact generation. Domain compositions identified are nucleotidylcyclase III, BLUF, NIT, P-loopNTPase, HAMP and Cache1

Several orthogroups consist of an expansive cohort of *E. gracilis* sequences, and a selected few were analyzed phylogenetically and annotated for protein architectural/domain features (Additional file 1: Figure S5, Additional file 4: Table S3). Firstly, highly significant in terms of size and evolutionary history is a family of nucleotidylcyclase III (NCIII)-domain-containing proteins widely distributed across eukaryotes. In African trypanosomes, adenylate cyclases are mediators of immune modulation in the mammalian host [71]. One nucleotidylcyclase subfamily is restricted to kinetoplastids and organisms with secondary plastids and contains photosensor adenylate cyclases [12] that possess one or two BLUF domains (blue light sensor) with a double NCIII domain (Fig. 4). These nucleotidylcyclases are phylogenetically similar to the NCIII-family of *N. gruberi* [66]. A second subfamily is pan-eukaryotic and possesses one NCIII domain and several *trans*-membrane domains, a HAMP (histidine kinases, adenylate cyclases, methyl-accepting proteins and phosphatases) domain as well as cache 1 (calcium channel and chemotaxis receptor) domains. These domains are associated with proteins involved, as their name implies, in signal transduction, particularly chemotaxis [76, 77]. Again, this subfamily is closely related to NCIII-family genes from *N. gruberi*. The third subfamily represents a kinetoplastid cluster with *trans*-membrane proteins and frequently also HAMP and cache1 domains. This complexity indicates considerable flexibility in nucleotidylcyclase evolution and that many lineage-specific paralogs have arisen, with implications for signal transduction, suggesting an extensive regulatory and sensory capacity in *E. gracilis*.

A second example is a large protein kinase C-domain containing a group of protein kinases, which also exhibit extensive lineage-specific expansions in *E. gracilis* (several orthogroups contained a very large number of *E. gracilis* sequences, and a few selected were analysed phylogenetically and annotated for architecture (Additional file 1: Figure S5)). A third orthogroup possess a signal receiver domain (REC) with clear lineage-specific *E. gracilis* paralogs present (Additional file 1: Figure S5). The *E. gracilis* members possess an H-ATPase domain, which is distinct from the Per-Arnt-Sim (PAS) domain present in many orthologs from other lineages. The presence of independently expanded signaling protein families in *E. gracilis* suggests both highly complex and divergent pathways. These very large families likely partly explain the expanded coding potential in *E. gracilis*, as well as provide some indication of how sensing and adaptation to diverse environments is achieved.

### Conservation and divergence of systems between *E. gracilis* and kinetoplastids

To better understand the evolution of *Euglena* and its relationship to free living and parasitic relatives, we selected multiple cellular systems for detailed annotation. These were selected based on documented divergence between kinetoplastids and other eukaryotic lineages and encompass features of metabolism, the cytoskeleton, the endomembrane system and others (Additional file 5: Table S4). Additional annotations of systems not discussed here are available in Additional file 5: Table S4 and provided in Additional file 6: Supplementary analysis.

A unique feature of energy metabolism in kinetoplastids is compartmentalisation of several glycolytic enzymes within peroxisome-derived glycosomes and the presence of additional enzymes for metabolism of the glycolytic intermediate phospho-enolpyruvate to succinate [78]. Glycosomes have been recently reported in diplonemids, the second major euglenozoan group, suggesting an origin predating kinetoplastida [79]. Using 159 query protein sequences for experimentally supported glycosomal *T. brucei* proteins [80], we found candidate orthologs for the majority, but based on the absence of detectable PTS-1 or PTS-2 targeting signals, no evidence that enzymes linked to carbohydrate metabolism are (glyco)peroxisomal. Of the 159 queries, 49 are annotated as hypothetical or trypanosomatid-specific and none had a detectable ortholog in *E. gracilis* (Additional file 5: Table S4). Collectively, this suggests that peroxisomes in *E. gracilis* most likely function in diverse aspects of lipid metabolism rather than glycolysis or other aspects of carbohydrate metabolism and distinct from kinetoplastids.

The surface membrane of *E. gracilis* is in close association with a microtubule corset, and with some structural similarity to the subpellicular array of trypanosomatids, but with very unique architecture [81]. While the plasma membrane composition of kinetoplastids is lineage-specific, in terms of many major surface proteins and a major contributor to host-parasite interactions [82], transporters and some additional surface protein families are more conserved. To compare with *E. gracilis*, we predicted membrane proteins using the signal peptide together with orthogroup clustering, which will encompass both surface and endomembrane compartment constituents. Many genes have significant similarity to kinetoplastids (1103), *B. saltans* (32) or non-kinetoplastida (487) (Additional file 7: Table S5). About 698 proteins with a signal peptide appear to be *E. gracilis* specific, and most of these are a single copy (87.5%), while there are clear large families that possess conserved features (see above). Notably, we were unable to identify a rhodopsin homolog, in contrast to several biochemical analyses suggesting the presence of retinal, the rhodopsin cofactor, which has been interpreted as evidence for a rhodopsin-like light sensor. It remains possible that the euglenid rhodopsin was not represented in the transcriptome or is too divergent to detect [83].

In common with *B. saltans*, *E. gracilis* has a distinct class of amastin, a major kinetoplastid surface protein and which arose from a single ancestor shared with the last euglenozoan common ancestor (Additional file 1: Figure S6). *E. gracilis* also possesses enzymes for the synthesis of lipophosphoglycan (LPG), a glycoconjugate first described in *Leishmania* and implicated in defense and disease mechanisms, together with the pathways for synthesis of GPI protein anchors and free lipids. These data suggest that LPG predates the evolution of parasitism and that the ancestral role was possibly more general, for example, a defense against proteases or predation, or in cell-cell/cell-substrate interactions. Significantly, gp63, a major surface protein present in the vast majority of eukaryotes and also involved in *Leishmania* pathogenesis, is absent and represents a secondary loss following separation from the kinetoplastid lineage.

The endomembrane system is responsible for biosynthesis, degradation and targeting of proteins and lipids and can be considered as a proxy for intracellular complexity. Compartments and transport routes can be predicted with accuracy based on the presence of genes encoding proteins mediating these routes. Using such an analysis, it has been predicted that the complexity of endomembrane compartments in trypanosomatids is decreased compared with free-living bodonids [23, 84]. *E. gracilis* possesses a relatively complete set of membrane-trafficking proteins, extending this trend further (Additional file 1: Figure S7). Two key adaptin family complexes involved in vesicle coat formation and post-Golgi transport, AP5 and TSET, are absent from kinetoplastids, and while AP5 is also absent from *E. gracilis*, a near complete TSET is present. Significantly, endosomal pathways are predicted as more complex than kinetoplastids, with multiple Rab7 (late endosome/lysosome) and Rab11 (recycling endosome) paralogs, together with ER-associated paralogs for Rab1 (early anterograde transport) and Rab32, respectively. Rab32 may also be associated with the contractile vacuole, an endolysosomal organelle responsible for osmoregulation in many freshwater protists, but these aspects of *E. gracilis* biology remain to be explored.

In kinetoplastids, an unusual cytoskeletal element, the bilobe, plays a central role in Golgi, flagellar pocket collar and flagellum attachment zone biogenesis [74]. All of the structural proteins (MORN1, RRP1, BILBO1, Centrin-2 and Centrin-4) were found [85–90] (Additional file 5: Table S4). Therefore, the potential for the synthesis of a bilobe-like structure in *E. gracilis* is supported, although clearly experimental evidence is needed for the presence of such a structure, but which suggests an origin predating the kinetoplastids.

The considerable size of the *E. gracilis* genome and complex splicing patterns suggests the presence of sophisticated mechanisms for organizing chromatin, mRNA processing and transcription [53, 57]. Furthermore, the *E. gracilis* nucleus has somewhat unusual heterochromatin morphology, with electron-dense regions appearing as numerous foci throughout the nucleoplasm (Additional file 1: Figure S8). Nucleoskeletal proteins related to lamins, NMCPs of plants or kinetoplastid-specific NUP-1/2 are all absent from *E. gracilis*, suggesting that anchoring of chromatin to the nuclear envelope exploits a distinct mechanism [91]. Further, while much of the nuclear pore complex (NPC) is well conserved across most lineages, orthologs for DBP5 and Gle1, two proteins involved in mRNA export in mammalian, yeast and plant NPCs, but absent from trypanosomes, are present. This is consistent with an earlier proposal that the absence of DBP5/Gle1 is connected to the loss of *cis*-splicing in kinetoplastids, but indicates that this is not due to the presence of *trans*-splicing per se as this is common to *E. gracilis* and the kinetoplastids [92]. Finally, kinetochores, required for engagement of chromosomes with the mitotic spindle, are also highly divergent in trypanosomes (Additional file 1: Figure S8) [93, 94]. Of the trypanosomatid kinetochore proteins, only KKT19 and KKT10 are obviously present in *E. gracilis*; as these are a kinase and phosphatase, respectively, they may not be bona fide kinetochore proteins in *E. gracilis*. Further, very few canonical kinetochore proteins were found, suggesting possible divergence from both higher eukaryote and trypanosome configurations. Overall, these observations suggest unique mechanisms operate in the *E. gracilis* nucleus, which may reflect transitions between conventional kinetochores, lamins and nuclear pores into the more radical configuration present in kinetoplastids. Additional systems are discussed in supplementary material (Additional file 6).

### The *Euglena* mitochondrion

In kinetoplastids, unique mitochondrial genome structures are present [95]. Typically, kinetoplastid mitochondrial genomes comprise ~ 40 copies of a maxicircle encoding several mitochondrial proteins and several thousand minicircles encoding guide RNAs for editing maxicircle transcripts [40, 95]. In trypanosomatids, this structure is attached to the flagellum basal body via a complex cytoskeletal element, the tri-partite attachment complex (TAC) [95]. We find no evidence for RNA editing in *E. gracilis*, nor for the TAC, both of which are consistent with the presence of a mitochondrial genome composed of only short linear DNA molecules and a conventional mitochondrial mRNA transcription system [39]. Specifically, only 16 of 51 proteins involved in RNA editing in *T. brucei* [96] had reciprocal best BLAST hits, and only one predicted protein contained a mitochondrial targeting signal. No homologs to TAC proteins were found (Additional file 5: Table S4).

The *E. gracilis* mitochondrial proteome is predicted to exceed 1000 proteins and encompasses 16 functional categories (Additional file 1: Figure S9A). The kinetoplastid mitochondrion possesses a non-canonical outer mitochondrial membrane translocase (A)TOM (archaic translocase of the outer membrane). The major component is (A)TOM40, a conserved beta-barrel protein that forms the conducting pore, but which is highly diverged in kinetoplastids [97–99]. We identified homologs of two specific receptor subunits of (A)TOM, namely ATOM46 and ATOM69 [100], and two TOM40-like proteins; both these latter are highly divergent and could not be assigned unequivocally as TOM40 orthologs.

**Fig. 5:**
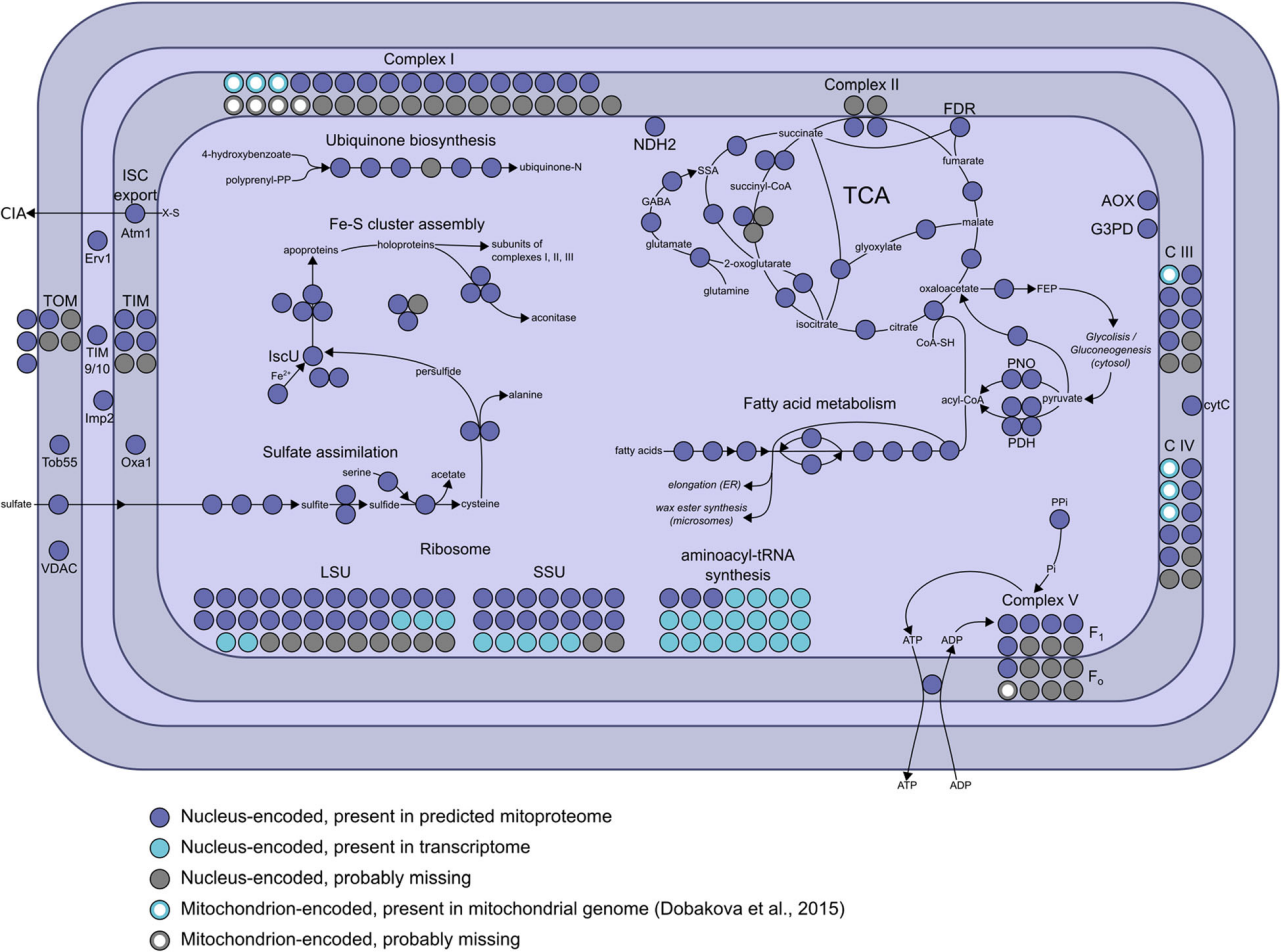
*Euglena gracilis* has flexible and fault-tolerant mitochondrial metabolism. Proteins involved in mitochondrial pathways and complexes are shown, including: tricarboxylic acid (TCA) cycle, pyruvate dehydrogenase, fatty acid metabolism, complexes I-V of respiratory chain, ubiquinone biosynthesis, sulfate assimilation pathway, Fe-S cluster assembly and export, TIM/TOM complex and mitochondrial import machinery. Colour codes: dark blue, nucleus encoded, present in predicted mitochondrial proteome; light blue, present in transcriptome without evidence for mitochondrial localization; light blue/white, mitochondrion-encoded proteins identified previously [39]; grey, expected in nuclear transcriptome and not found; grey/white, expected in mitochondrial genome and not found. The *E. gracilis* mitochondrion can produce energy under both aerobic and anaerobic conditions and has workarounds for the main mitochondrial pathways, such as TCA cycle and respiratory chain, which may in part explain the outstanding adaptability of this organism

We also identified canonical subunits of respiratory chain complexes I–V and 27 homologs of kinetoplastid-specific proteins, together with the widely represented alternative oxidase, consistent with earlier work [101]. Moreover, an ortholog of *T. brucei* alternative type II NADH dehydrogenase (NDH2) was detected. We found only 38 of 133 canonical and only three of 56 kinetoplastid-specific mitoribosomal proteins, which suggests considerable divergence. Hence, the *E. gracilis* mitochondrion has unique features, representing an intermediate between the mitochondria familiar from yeast or mammals and the atypical organelle present in kinetoplastids (Fig. 5).

### The *Euglena* plastid

**Fig. 6:**
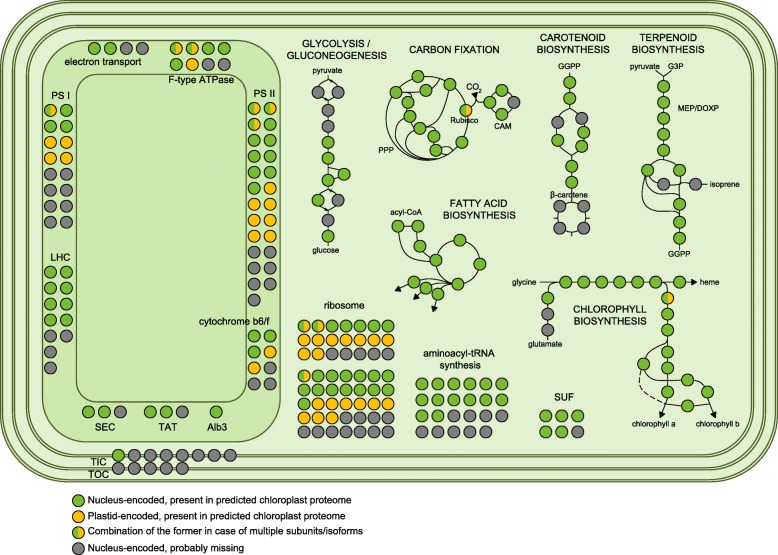
The *Euglena gracilis* plastid possesses broad metabolic potential. Proteins involved in core plastid metabolic pathways were identified and include glycolysis/gluconeogenesis, carbon fixation, fatty acid biosynthesis, carotenoid biosynthesis, isoprenoid biosynthesis, and chlorophyll biosynthesis. Colour codes: green, nucleus encoded, present in predicted chloroplast proteome; amber, plastid encoded, present in predicted chloroplast proteome; light green/white, combination of green and amber in case of multiple subunits/isoforms; and gray, expected but not found

The *Euglena* chloroplast, as a secondary acquisition, represents a near unique configuration for studying fundamental aspects of organelle origins and evolution. The predicted *E. gracilis* plastid proteome contains 1902 proteins (Fig. 6, Additional file 1: Figure S9B; Additional file 8: Table S6). Typical plastid metabolic pathways and enzymes are present, including 70 proteins involved in the chloroplast electron transport chain and light harvesting antennae. A few expected genes were absent, such as glycolytic glucose-6-phosphate isomerase and carotenoid synthesis 15-*cis*-phytoene desaturase; as both pathways are known to be present, these likely arise from incomplete sequence data [41]. The C_5_ tetrapyrrole pathway was completely reconstructed, while the C_4_ pathway for aminolevulinate synthesis is absent, consistent with previous findings [102]. Enzymes connecting the cytosolic/mitochondrial mevalonate and plastid methyl-d-erythritol pathway (MEP/DOXP) pathways of terpenoid synthesis were not found, in accordance with separate plastid and cytosolic pools of geranylgeranyl pyrophosphate. Carotenoid and non-plastid isoprenoid (e.g. sterols, dolichols) biosynthetic pathways appear unconnected [103]. Significantly, over 50% of the predicted plastid proteome represent proteins with no homology in the databases, suggesting considerable novel metabolic potential.

Protein targeting to the *E. gracilis* plastid involves trafficking via the Golgi complex. Since the plastid was newly established in the euglenoid lineage, this implies that at least two novel membrane-trafficking pathways should be present, one anterograde *trans*-Golgi to plastid and a retrograde pathway operating in reverse. The relevant machinery for such pathways could be produced via either gene transfer from the green algal host or duplication of host membrane-trafficking machinery. We found no reliable evidence for contributions to the endomembrane protein complement by endosymbiotic gene transfer, but there are extensive gene duplications within the endomembrane machinery. Specifically, additional paralogs of key factors involved in post-Golgi to endosome transport, e.g. AP1 and Rab14, are present, as are expansions in retromer and syntaxin16 that specifically serve to retrieve material from endosomes to the *trans*-Golgi network. Overall, we suggest both a period of kleptoplasty prior to stable establishment of the secondary green plastid and a model whereby novel transport pathways were established by gene duplication, as proposed by the organelle paralogy hypothesis [44].

## Conclusions

We present here a detailed analysis of the protein-coding complement of *E. gracilis*, together with insights into genome organization. The genome is very large for a unicellular organism, consistent with many earlier estimates and has exceptionally low coding content, similar to large metazoan genomes. BUSCO, CEGMA and also annotation of many metabolic pathways, complexes and systems indicate that both our data and that from previous work attained very high coverage of the transcriptome. Significantly concatenation of all three datasets resulted in essentially negligible improvement to BUSCO scores, suggesting that the data approach a complete sampling.

We predict a highly divergent surface proteome with expanded signal transduction capabilities likely present at the plasma membrane. *E. gracilis* possesses machinery for synthesis of lipophosphoglycan, suggesting the presence of a defensive phosphoglycan sheath [104]. Significantly, we find evidence for gradual loss of conventional kinetochores, *cis*-splicing and complex RNA processing at the NPC during Euglenozoa evolution. Unexpectedly, there is little evidence for transcriptional control, highly similar to kinetoplastids. Reliance on post-transcriptional processes has been recognized as a feature of *E. gracilis* [105] with mounting evidence that translational and degradative processes are crucial determinants of protein abundance and in agreement with this work [106]. An extensive endomembrane system indicates complex internal organization and multiple endosomal routes representing mechanisms for the sorting, uptake and digestion of material from a range of sources. We also find evidence for novel trafficking pathways between the endomembrane system and the chloroplast; this, together with analysis of the nuclear genome and likely origins of many genes, provides insights into the processes by which secondary plastids become enslaved, and is consistent with a protracted period of plastid acquisition.

## Materials and methods

### Cultivation

*E. gracilis* strain Z1 was provided by William Martin (Düsseldorf). Cells were cultivated at ambient temperature under continuous illumination from a 60-W tungsten filament bulb at 20 cm from the culture vessel, in Hutner’s media [107]. Cells were collected in exponential growth phase at ~ 9 × 10^5^ cells/ml, measured using a haemocytometer. For light and dark adaptation, cells were adapted to Hutner heterotrophic medium [107] for 16 days prior to the initiation of a light or dark growth period. Cultures were subcultured and dark-adapted cultures transferred to a light proof box adjacent to the light cultures. Subculturing was done under low light conditions periodically and cultures maintained for up to 2 weeks prior to harvesting. The impact of a prolonged period under dark conditions was assessed by microscopy (Zeiss LSM 700 confocal microscope; × 40 Plan-Neofuar NA1.3 lens under phase contrast, by UV/VIS spectroscopy using a Shimadzu UV-2450, wavelength scan of 190–800 nm and SDS-PAGE).

### Isolation of RNA and proteins for gene expression studies

Equivalent numbers (1 × 10^7^ cells) of dark or light cultured cells were harvested by centrifugation at 25 °C, 1000*g* for 10 mins. RNA extraction was performed using the Qiagen RNeasy Mini Kit (Cat. No. 74104). Genomic DNA contamination was eliminated by performing on-column DNase digestion. Extracted RNA was preserved at − 80 °C for RNA sequencing. For proteomics, cells were washed with PBS containing complete protease inhibitors (Roche), extracted with NuPAGE sample buffer (3X), sonicated and lysates containing 1 × 10^7^ cells fractionated on a NuPAGE Bis-Tris 4–12% gradient polyacrylamide gel (Thermo Scientific, Waltham, MA, USA) under reducing conditions. The sample lane was divided into eight slices that were subjected to tryptic digestion and reductive alkylation.

### Proteomics analysis for gene expression studies

Liquid chromatography tandem mass spectrometry (LC-MS^2^) was performed in house at the University of Dundee, UK. Samples were analyzed on a Dionex UltiMate 3000 RSLCnano System (Thermo Scientific, Waltham, MA, USA) coupled to an Orbitrap Q-exactive mass spectrometer (Thermo Scientific) at the University of Dundee proteomics facility. Protein mass spectra were analyzed using MaxQuant version 1.5 [108] searching the predicted *E. gracilis* proteome from the de novo transcriptome assembly reported here. Minimum peptide length was set at six amino acids, isoleucine and leucine were considered indistinguishable and false discovery rates (FDR) of 0.01 were calculated at the levels of peptides, proteins and modification sites based on the number of hits against the reversed sequence database. Ratios were calculated from label-free quantification intensities using only peptides that could be uniquely mapped to a given protein. If the identified peptide sequence set of one protein contained the peptide set of another protein, these two proteins were assigned to the same protein group. *P* values were calculated applying *t* test-based statistics using Perseus [109]. There were 8661 distinct protein groups identified by MaxQuant analysis. For further analyses, data were reduced to 4297 protein groups by rejecting those groups not identified at the peptide level in each of the three replicates for one state. Additionally, a cohort of 384 protein groups was extracted that were observed in only one state (232 light and 152 dark).

### Ultrastructure of *E. gracilis* cells in light and dark conditions

Two populations of *E. gracilis* cells cultured in either light or dark conditions were initially fixed using 2.5% glutaraldehyde and 2% paraformaldehyde in 0.1 M sodium cacodylate buffer pH 7.2. Both samples were post-fixed for an hour in buffered 1% (*w*/*v*) OsO_4_ and embedded in molten agarose prior to incubating overnight in 2% (*w*/*v*) uranyl acetate. Agarose pellets were dehydrated through a graded acetone series and slowly embedded in Low Viscosity resin (TAAB Ltd.) over 4 days. Following polymerization, 70–90-nm-thin sections were cut by ultramicrotome, post-stained using 2% (*w*/*v*) uranyl acetate and Reynolds lead citrate [110] and imaged with a Hitachi H-7650 transmission electron microscope. Image resolution varied between 20 and 0.3 nm per pixel, depending on the magnification.

### Transcriptome analysis for gene expression studies

Extracted RNA was sequenced at the Beijing Genomics Institute (https://www.bgi.com/global/). Analysis and comparisons of the data were performed using standard pipelines. An estimated 62 M clean reads were generated which were subject to quality filtering using Trimmomatic [111], to remove low-quality bases and read pairs as well as contaminating adaptor sequences, prior to assembly. Sequences were searched for all common Illumina adaptors and settings for read processing by Trimmomatic were LEADING:10 TRAILING:10 SLIDINGWINDOW:5:15 MINLEN:50. The trimmed filtered reads were then used to quantify the de novo-assembled transcriptome using Salmon [112] with the bias-correction option operating. Expected counts were integerised before being subject to differential expression testing using DESeq2 [113] using default parameters. In the transcriptomics analysis, 66,542 distinct sequence classes were detected and the data was reduced to 41,045 applying the same rejection criteria as the proteome (minimum three replicates).

### Nucleic acid isolation and purification for genomic and transcriptomic studies

*E. gracilis* genomic DNA was isolated using the Qiagen DNA purification system to obtain low and high molecular weight DNA for Illumina paired-end and mate-pair read libraries (100-bp paired-end libraries with insert sizes of 170 bp, 500 bp and 800 bp, and mate-pair libraries with insert sizes of 2 kbp, 5 kbp and 40 kbp). For the shorter length libraries (≤ 5 kbp), cells were harvested by centrifugation for 10 mins at 1000 g and DNA extracted using the Qiagen DNAeasy blood and tissue kit (Qiagen Inc., Cat.No. 69504). The cultured animal cell protocol was modified and involved firstly, using 1 × 10^7^ cells, and secondly, prior to adding Buffer AL, 200 μl of RNase A was added to eliminate RNA contamination. Immediately after the washing step with Buffer AW2, centrifugation was performed for 1 min at 20,000*g* to eliminate traces of ethanol. To obtain high molecular weight DNA fragments for the ≥ 40 kb insert size library, the Qiagen Genomic-DNA isolation kit (blood and cell culture DNA kit - Maxi, Cat. No. 13362) was used. In this case, 1 × 10^8^ cells were harvested. Prior to adding Buffer C1, samples were ground in liquid nitrogen using a planetary ball mill (Retsch) [114] at 300 rpm for 3 min (the grinding was limited to two cycles to minimize DNA shearing). Four wash steps were performed to remove contaminants including traces of RNA. To determine molecular weight, 400 ng of DNA was loaded onto a 0.45% agarose gel in TAE buffer, stained with Thermo Scientific 6X Orange Loading Dye, and electrophoresed at 80 V for 2 h. A NanoDrop spectrophotometer (DeNovix DS-11+) was used to determine concentration and purity. Total RNA from *E. gracilis* was isolated using the Qiagen RNeasy Mini kit (Cat. No. 74104), and the protocol for the purification of total RNA from animal cells using spin technology was employed as above.

### Library preparation and sequencing for genomic and transcriptomic studies

Genome and transcriptome library preparation and sequencing were performed at the Beijing Genomic Institute, using Illumina Genome Analyzer HiSeq2000 and MiSeq. In the former case, paired-end genomic sequence of multiple read lengths (49 bp and 100 bp) corresponding to eight insert size libraries (170 bp, 250 bp, 500 bp, 540 bp, 800 bp, 2 kbp, 5 kbp, and 40 kbp) were generated with a combined length of ~ 57 Gbp. Additional PacBio libraries were generated at the University of Seattle (5.5 Gbp combined length) and Université Paris-Sud (3.3 Gbp combined length), and the data were kind gifts. A combined total of 305,447 PacBio circular consensus reads (CCS) were generated with estimated average length of 8870 bases and estimated coverage of ~ 1X.

### Genome and transcriptome assembly

Multiple routes were explored for the generation of an acceptable assembly [48]. The most successful strategy, as assessed by core eukaryotic gene mapping analysis (CEGMA) and the proportion of RNAseq reads that mapped to the genome assembly [115, 116], utilised Platanus [117], SSPACE [118] and String Graph Assembler (SGA) [119]. Here, the two MiSeq paired-end read libraries (150 bp paired-end and 300 bp paired-end libraries) and 100 bp (170 bp insert size) paired-end HiSeq read libraries were used for the Platanus assembly. Each of the paired-end read libraries was subject to overlapping paired-end read joining using the ErrorCorrectReads.pl algorithm of the ALLPATHS assembly package [120]. This step in ALLPATHS reduces the complexity of the input data by combining overlapping paired-end reads into single larger reads and performs well on independent benchmark tests of real and simulated data [120]. No other steps in the ALLPATHS assembly algorithm were used. These joined paired-end reads were provided to Platanus as single-end reads. The 500 bp and 800 bp insert size read libraries, which could not be subject to read joining as their insert sizes were too large, were included as single-end reads. This collective set of reads was provided to Platanus, and the method was run using its default parameters. The combined Illumina read data provided an estimated 25x coverage of the single-copy component of the genome by k-mer spectrum analysis using ALLPATHS (Additional file 1: Fig. S11). The resulting contigs from the Platanus [117] assembly were subject to six rounds of scaffolding and gap filling using the SSPACE [118] and SGA [119] algorithms. SSPACE was run with the following settings –a 0.7 –m 30 –n 50 –o 20 using the 500 bp and 800 bp insert size paired-end read libraries and the 2000 bp, 5000 bp and 40,000 bp insert size mate pair read libraries. Following each round of scaffolding, SGA was run on the scaffolds in gap filling mode (“-gapfill”) using the same combined input read library as Platanus above. This resulted in a de novo assembly with an N_50_ of 955 bp, comprising 2,066,288 scaffolds (Table S1).

A k-mer spectrum for the genome was calculated from the highest coverage read library (150 bp paired-end read library). It generated a single peak at 8.8× coverage, corresponding to the homozygous single-copy portion of the genome (Additional file 1: Figure S11A). Assuming a Poisson distribution that would be observed if all regions of the genome were single copy and homozygous, the estimated genome size of the single-copy proportion of genome is 487.2 Mb and the estimated size of the whole genome 2.33 Gb. The discrepancy between the Poisson model and the observed corresponds to multi-copy sequences, with a large proportion of low to medium copy number sequences represented at high frequency. There are more than 80,000 unique k-mers of length 31 that appear more than 10,000 times. These high copy number repeat sequences are those we refer to in the results and are most likely responsible for the difficulty with progressing an assembly further than we have been able to achieve.

To estimate the genome size and the proportion of the genome that is comprised of repetitive unique sequence a k-mer spectrum analysis was conducted (Additional file 1: Figure S11A). The largest Illumina paired-end read library (150-bp paired-end) was used for this analysis. Canonical k-mers were counted using jellyfish (Marçais et al. Bioinformatics 27(6): 764–770) at a range of different k-mer sizes (19, 21, 27 and 31). The resulting k-mer count histograms were analysed using GenomeScope [121]. Using these methods the haploid genome size was estimated to be between 330 mb and 500 mb (Additional file 1: Figure S11A). The repetitive component of the genome was estimated to be between 191 and 339 mb, and the unique component of the genome was estimated to be 141 mb to 160 mb (Additional file 1: Figure S11A). Heterozygosity was estimated to be between 2.2 and 2.6%.

The transcriptome assembly was generated by combining multiple different read libraries into a single transcriptome assembly. These included two 100 bp paired-end read libraries generated on an Illumina HiSeq2500 (200 bp insert size) that were previously published in [17]. *Euglena* transcriptome (PRJEB10085, 17) and the six 100-bp paired-end read libraries (200 bp insert size) were generated on an Illumina HiSeq2000 generated in this study (Additional file 2: Table S1, PRJNA310762). These read libraries were combined to give a total of 2.05 × 10^8^ paired-end reads that were provided as input for transcriptome assembly. Illumina adaptors and low-quality bases were trimmed from the reads using Trimmomatic. Ribosomal RNA sequence was removed using SortMeRNA [122] using default settings, before read error correction using BayesHammer [123] with default settings. Reads were normalized using khmer [124] with settings –C 20 –k 21 –M 8e9, and overlapping paired-end reads joined using ALLPATHS-LG [120] and all reads subject to de novo assembly using SGA, minimum overlap size of 80 nucleotides, no mismatches. These filtered, normalized, and joined reads were then mapped to this assembly using Bowtie2 [125]. Reads that were absent from the assembly were identified and placed with the assembled contigs into a new input file. This file containing the unassembled reads and assembled contigs was subject to assembly using SGA with an overlap size of 70. This process of identifying unmapped reads and reassembling with SGA was repeated each time, decreasing the overlap size by 10 nucleotides until a minimum overlap size of 40 was reached. This strategy was taken to minimize the occurrence of assembly errors that are commonly obtained when a default small k-mer size is used in de Bruijn graph assembly. Contigs were then subject to scaffolding using SSPACE and the full set of non-ribosomal, corrected, normalized paired-end reads using the settings –k 10, −a 0.7, −n 50, −o 20. Scaffolds were subject to gap filling using the SGA gap filling function. Finally, the assembled contigs were subject to base-error correction using Pilon [126] with the default settings. CEGMA [58] suggests ~ 88% completeness in terms of representation of coding sequence.

### Genome and transcriptome structural and functional automatic annotation

In silico analysis such as open reading frame (ORF) determination, gene predictions, gene ontology (GO) and KEGG (biological pathways) and taxa distribution were performed as part of an automatic functional annotation previously described [127] with minor modifications. Six frame translation and ORF determination of assembled transcriptome sequences were predicted using TransDecoder prediction tool [61] and Gene MarkS-T [128], and the longest ORF with coding characteristics, BLAST homology, and PFAM domain information extracted [129]. The predicted ORF was queried against the NCBI non-redundant protein database using BLASTp homology searches, and the top hit for each protein with an *E* value cutoff < 1e^−10^ retained. Using the Blast2GO automatic functional annotation tool [130], the GO annotations of the best BLAST results with an *E* value cutoff < 1e^−10^ were generated from the GO database. The protein domain, biological pathway analyses, and top species distributions were determined using InterPro, BLAST, enzyme code and KEGG [131]. To greatly reduce run times, BLASTp and Interpro scans were processed locally prior to uploading to Blast2GO in .xml file formats.

### Assembling sequence data, data mining and phylogenetic inference

Homology searches for orthologs and paralogs of specific biological annotations were performed against the predicted proteome for *E. gracilis* using BLASTp. Clustering at 100% identity was performed for the predicted *E. gracilis* proteins using the Cluster Database at High Identity (CD-HIT) [62] algorithm to remove gapped/incomplete and redundant sequences. Sequences with significant BLASTp top hit search (E value = 1e^−10^) were subjected to both Reversed Position Specific BLAST RPS-BLAST and InterProScan [132]. The annotated sequences with domain and/or protein signature matches were extracted using a combination of custom UNIX commands and BioPerl scripts and clustered to 99% identity using CD-HIT. CD-HIT outputs a set of ‘non-redundant’ (nr) protein representative sequences which were aligned to known eukaryotic protein reference sequences using ClustalX2 [133] and MAFFT [134]. Poorly aligned positions or gaps were removed using the gap deletion command prior to alignment, and the final alignments processed locally for phylogenetic inference with the PhyML Command Line Interface (CLI) using default settings [135], RAxML [136], FastTree [137] and MrBayes [138]. Annotations of the trees were performed using TreeGraph2 [139] and Adobe Illustrator (Adobe Inc.).

### Contigs > 10 kbp in the *E. gracilis* genome

For an initial insight into the architecture of the genome contigs > 10 kbp were analyzed. These contigs were interrogated using tBLASTn with the *E. gracilis* proteome predicted from the transcriptome. Sequences with hits were further interrogated using the Exonerate algorithm [59] for insights into splicing mechanisms and coding regions using the --protein2genome and --showquerygff and --showtargetgff options. Sequences, and their respective splicing coordinates in gff3, were uploaded to the Artemis genome viewer [140] for visualization. Coding regions in gff formats were extracted and translated using a combination of BEDtools getfasta [141] and the EMBOSS getorf [142] tools.

### Orthologous group clustering

To identify orthologous genes in *E. gracilis* shared across eukaryotic taxa, we clustered the *E. gracilis* predicted proteome with 30 selected eukaryotic taxa using OrthoFinder [70] with taxa distribution including kinetoplastids, other members of the excavates, unikonts, bikonts, green algae, land plants and red algae.

### Phylogenetic analyses of ancestry of *Euglena* genes

All 36,526 predicted nucleus-encoded proteins were searched (BLASTp 2.2.29) against a custom database containing 207 organisms (Additional file 3: Table S2). Homologues with *E* value < 10^−2^ were retrieved. Since an unrooted phylogenetic tree can be calculated only for three or more organisms, all proteins with less than three recovered homologues (16,636 proteins) were excluded. The remaining (19,890 proteins) were aligned (MAFFT 7.273; default parameters) and trimmed (trimAl 1.2 [143], default parameters). Alignments longer than 74 amino acid residues and with all sequences determined, i.e. there was no sequence containing only undetermined characters, (18,108 alignments) were used for tree reconstruction. The trees were calculated with RAxML [136] (v8.1.17; 100 rapid bootstraps) in Metacentrum (The National Grid Infrastructure in the Czech Republic). Custom scripts (Python 3.4) were used to sort the trees into bins based on the taxonomic affiliation of the clan in which *E. gracilis* branched. The tree was included in a bin if a bipartition supported by bootstrap 75% and higher comprised of *E. gracilis* and members of one defined taxonomic group only. In 34 cases, in which *E. gracilis* was contained in two such bipartitions containing taxa from different defined group, the tree was assigned to the two respective bins.

### Mitochondrial proteome prediction

The predicted proteins were subjected to Blast2GO [130] and KEGG automatic annotation server (KAAS [144]) automatic annotation, BLASTp searches against the *T. brucei*, *Homo sapiens*, *Saccharomyces cerevisiae* and *Arabidopsis thaliana* reference mitoproteomes and, finally, targeting signal prediction using TargetP [145]. *E. gracilis* protein was predicted as mitochondrial if (i) TargetP mitochondrial score was higher than 0.9 (607 proteins), or (ii) there was an ortholog in at least one reference mitoproteome, not associated with non-mitochondrial functions (343 proteins), or (iii) assigned mitochondrial by Blast2GO (with the exception of the MTERF family) (62 proteins). The missing members of the found mitochondrial pathways and modules were identified by a manual search (81 proteins). To streamline the final annotated output and to ensure retention of only the most reliable predictions, we chose the most confident annotation between Blast2GO, BLASTp and KAAS for each protein. The final mitochondrial dataset includes 1093 proteins.

### Plastid proteome prediction

The translated *E. gracilis* transcriptome (predicted proteome) was subjected to signal prediction pipeline using a combination of SignalP [146] and PrediSI [147] while chloroplast transit peptide prediction was performed using ChloroP [148]. The sequences which scored positive by either SignalP (2551 sequences) or PrediSI (4857 sequences) were cut at the predicted signal peptide cleavage site. The sequences were then truncated to maximum length of 200 amino acid residues for faster calculation and analyzed by ChloroP. The preliminary dataset of *E. gracilis* plastid targeted proteins (1679 sequences) consisted of transcripts which scored positive in SignalP + ChloroP (59 sequences), PrediSI + ChloroP (1002 sequences) and SignalP + PrediSI + ChloroP (618 sequences) analysis. In the second step, model dataset of 920 sequences of *Arabidopsis thaliana* proteins localized to the plastid envelope, stroma, thylakoid, grana and lamellae obtained from the public AT_CHLORO proteomic database [149] were searched by BLAST against the whole translated *E. gracilis* transcriptome and the identified orthologs were then combined with the results of orthogroup clustering performed by OrthoFinder (see above). Based on these searches, an additional 144 sequences representing orthologs of *A. thaliana* chloroplast proteins were added to the dataset of *E. gracilis*-predicted plastid proteome regardless of their targeting sequences. This enriched dataset of 1823 proteins was annotated automatically using BLAST at NCBI, KOBAS [150] and KAAS [144] independently. All automatic annotations including KO and EC numbers were then revised and edited or corrected manually and used for metabolic map reconstruction. The missing enzymes and subunits of otherwise chloroplast pathways and complexes were investigated and eventually added manually to the set regardless of their targeting sequences during the manual annotation and pathway reconstruction. This approach resulted in inclusion of another 79 sequences. The final set of predicted *E. gracilis* chloroplast proteins consisted of 1902 entries.

## Additional files

Additional file 1: **Figure S1.** Organisation of open reading frames in the *E. gracilis* genome. **Figure S2.** Functional analysis of *E. gracilis* coding capacity by Gene Ontology. Figure S3. Dark adapted cells have altered proteomes and transcriptomes. **Figure S4.** Orthogroup clusters in *E. gracilis* and selected eukaryotes. **Figure S5.** Phylogeny of selected shared large paralog families. **Figure S6.** Surface families of *E. gracilis*. Figure S7. The *E. gracilis* endomembrane system. **Figure S8.** The *E. gracilis* nuclear pore and kinetochore complexes. **Figure S9.** The predicted proteomes of *E. gracilis* organelles. **Figure S10.** Metabolism in *E. gracilis.* **Figure S11.** Additional assembly features. **Figure S12.** BUSCO comparisons between the present work and prior transcriptomes. (PDF 10993 kb)

Additional file 2: **Table S1.** Raw data for proteomics and transcriptomics of *E. gracilis* under adaptive conditions. Cells were grown under dark or light conditions as described in methods and subjected to protein or RNA extraction and analysed by mass spectrometry or RNAseq. Each condition was analysed in triplicate (*n* = 3) and data for individual samples together with the merged data are provided (Transcripts, Proteome), together with BLAST annotation of altered transcripts (additional tabs). (XLSX 19876 kb)

Additional file 3: **Table S2.** Analysis of phylogenetic relationships of *E. gracilis* proteins. The sheet contains three tables. First table summarizes the taxon composition of the custom database used for the search of homologues of *E. gracilis* proteins. Second table summarizes the number of items in each step and the pipeline. The third table gives exact numbers of trees that fell into defined taxonomic bins. (XLSX 16396 kb)

Additional file 4: **Table S3.** Analysis of GO term frequency, domains and large orthogroup architecture. Sheet 1: GO terms in orthogroups. The sheet has two subtables. In one the GO terms represented above 5% in each orthogroup are shown - all other GO terms with less than 5% frequency have been omitted as the numbers of sequences included are very small. The second shows the number of annotated and non-annotated sequences of each taxonomic group selected. Yellow highlight shows the GO terms of interest belonging to *molecular process* that are analyzed in this study. Sheet 2: Conserved domains from NCBI database (CDD) detected in those sequences with the GO terms of interest highlighted in sheet 1. Output provided by CDD searches. For the sequence identifiers, note that first field separated with “_”, represents the taxonomic group to which it belongs. Sheet 3: Incidence of conserved domains detected in CDD searches and orthogroups. This table summarizes the output of the CDD searches. Gray highlight represents the conserved domains in parallel with the respective orthogroup (OG number) of the sequences for which we provide phylogenetic analyses. Sheet 4: Data for annotation of NCIII tree. *Trans*-membrane domains and conserved domains. Sheet 5: Data for annotation of REC tree. *Trans*-membrane domains and conserved domains. (XLSX 127 kb)

Additional file 5: **Table S4.** Accessions of genes associated with specific cellular functions. Each worksheet contains details of the orthologs and their accession numbers for a specific subset of predicted ORFs associated with an indicated cellular function, metabolic process or organelle. The first two sheets show the overall predictions (all annotated transcripts) and a summary graphic (Distributions). (XLSX 870 kb)

Additional file 6: Supplementary analyses. (DOCX 17 kb)

Additional file 7: **Table S5.** Surface/endomembrane proteome predictions. Panel A: Predicted numbers of ORFs encoded in the *E. gracilis* predicted proteome that contain a signal sequence (SS) together with additional determinants for stable membrane attachment (i.e. a glycosylphosphatidylinositol anchor (GPI) or trans-membrane domain (TMD)). Panel B: Frequency distribution of predicted *Euglena*-specific surface gene families, shown as number of families according to size. 608 (87.5%). *Euglena*-specific surface genes are predicted to be single-copy, whereas five families are predicted to have more than seven members. Panel C: PHYRE 2.0 summary results for an element of each multi-copy family (*n* > 4) of *E. gracilis*, including family size, residues matching the model and correspondent coverage of the sequence, percentage identity, confidence of prediction, and description of top template model. (XLXS 44 kb)

Additional file 8: **Table S6.** Predicted proteomes for the *E. gracilis* plastid and the mitochondrion. Panels include summaries for each organelle for numbers of genes in functional categories found, annotations for transcripts predicted as mitochondrial or chloroplastic and finally a reconstruction of major mitochondrial complexes and pathways. (DOCX 141 kb)
