# Supplementary material for: Transcriptome, proteome and draft genome of Euglena gracilis
Source: BMC Biol. 2019 Feb 7;17:11. doi: 10.1186/s12915-019-0626-8 (PMC6366073; doi:10.1186/s12915-019-0626-8)
Supplement: Supplementary file 1 — Figure S1. Organisation of open reading frames in the E. gracilis genome. Figure S2. Functional analysis of E. gracilis coding capacity by Gene Ontology. Figure S3. Dark adapted cells have altered proteomes and transcriptomes. Figure S4. Orthogroup clusters in E. gracilis and selected eukaryotes. Figure S5. Phylogeny of selected shared large paralog families. Figure S6. Surface families of E. gracilis. Figure S7. The E. gracilis endomembrane system. Figure S8. The E. gracilis nuclear pore and kinetochore complexes. Figure S9. The predicted proteomes of E. gracilis organelles. Figure S10. Metabolism in E. gracilis. Figure S11. Additional assembly features. Figure S12. BUSCO comparisons between the present work and prior transcriptomes. (PDF 10993 kb) [file 12915_2019_626_MOESM1_ESM.pdf]

Fig S1

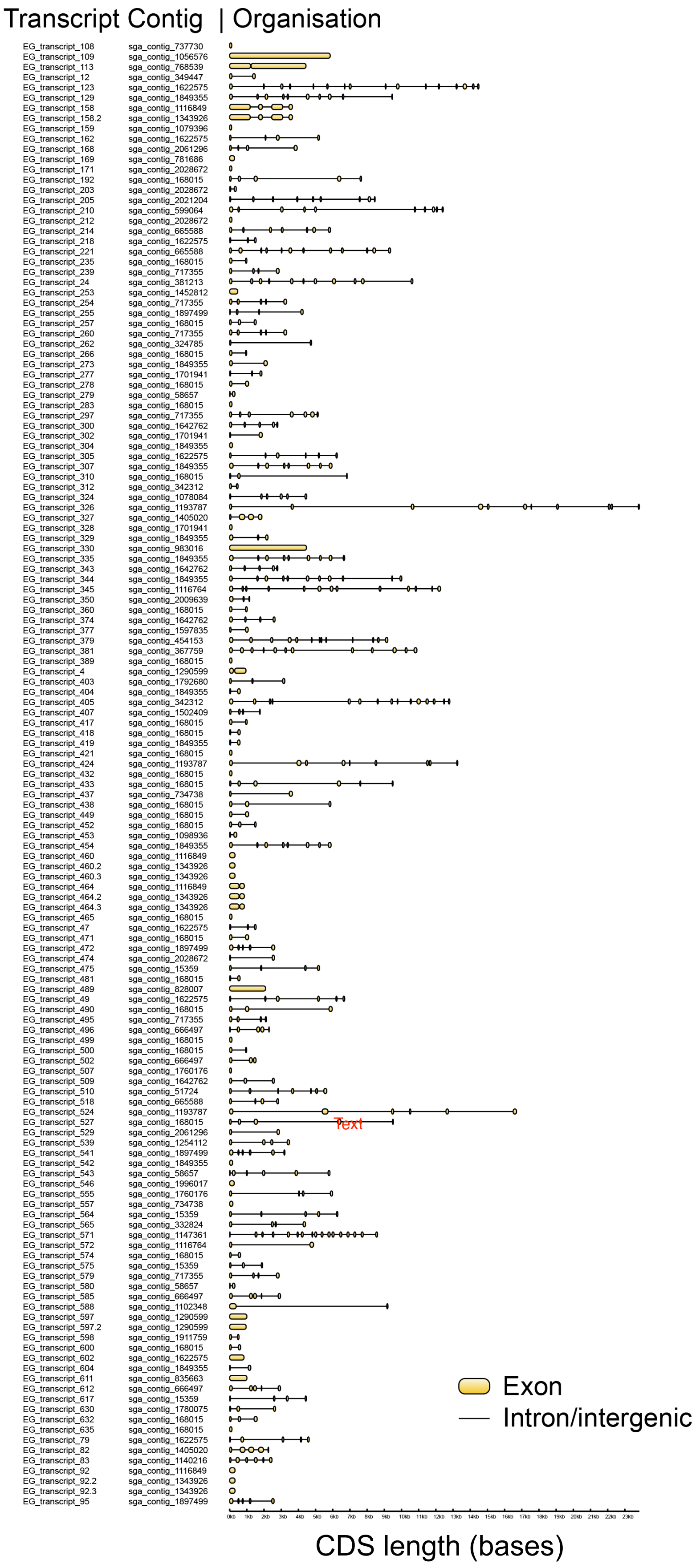

Figure S1: Organisation of open reading frames in the *E. gracilis* genome.

Fig S2

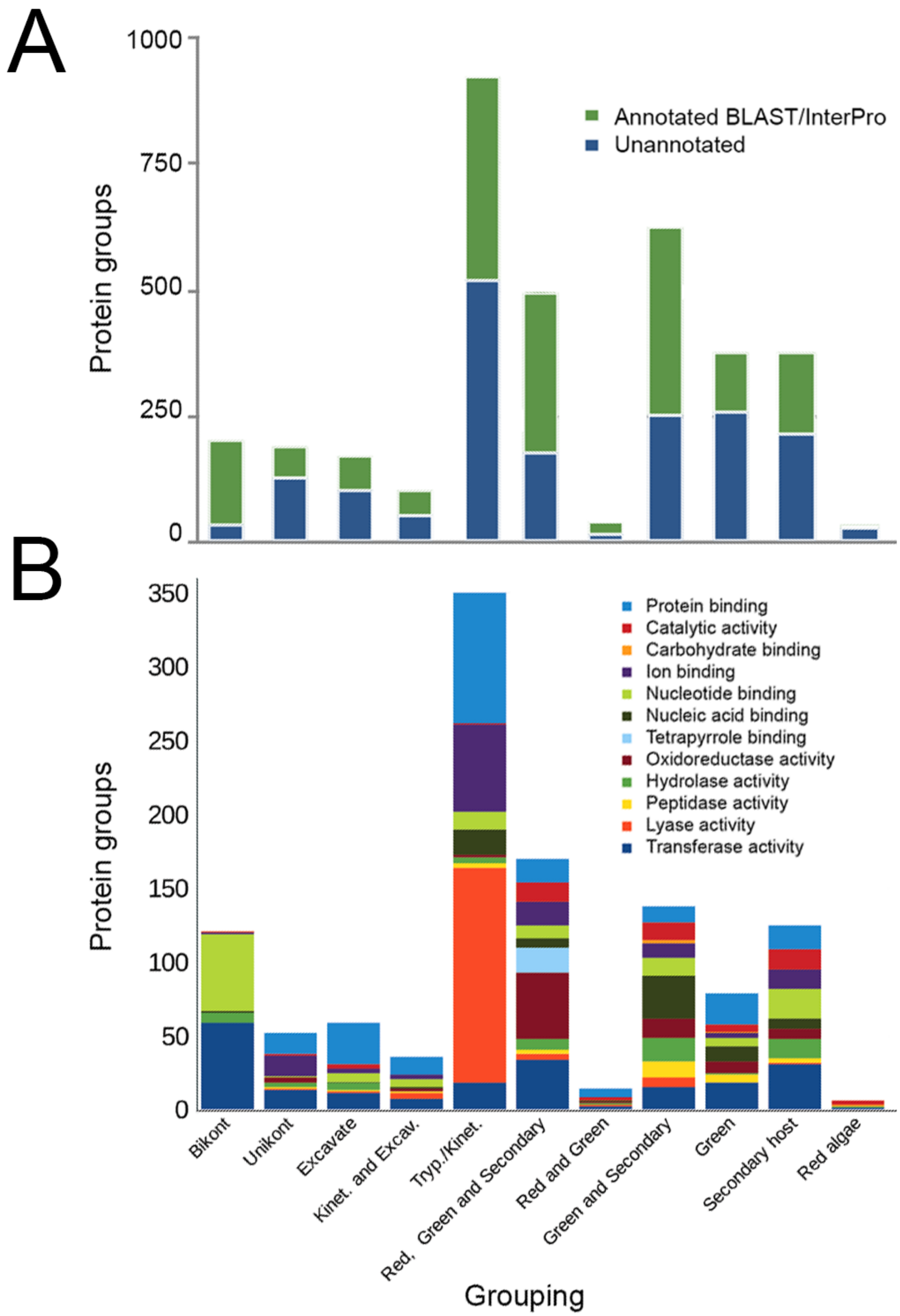

Figure S2: Functional analysis of Euglena coding capacity by Gene Ontology.

Fig S3A-F

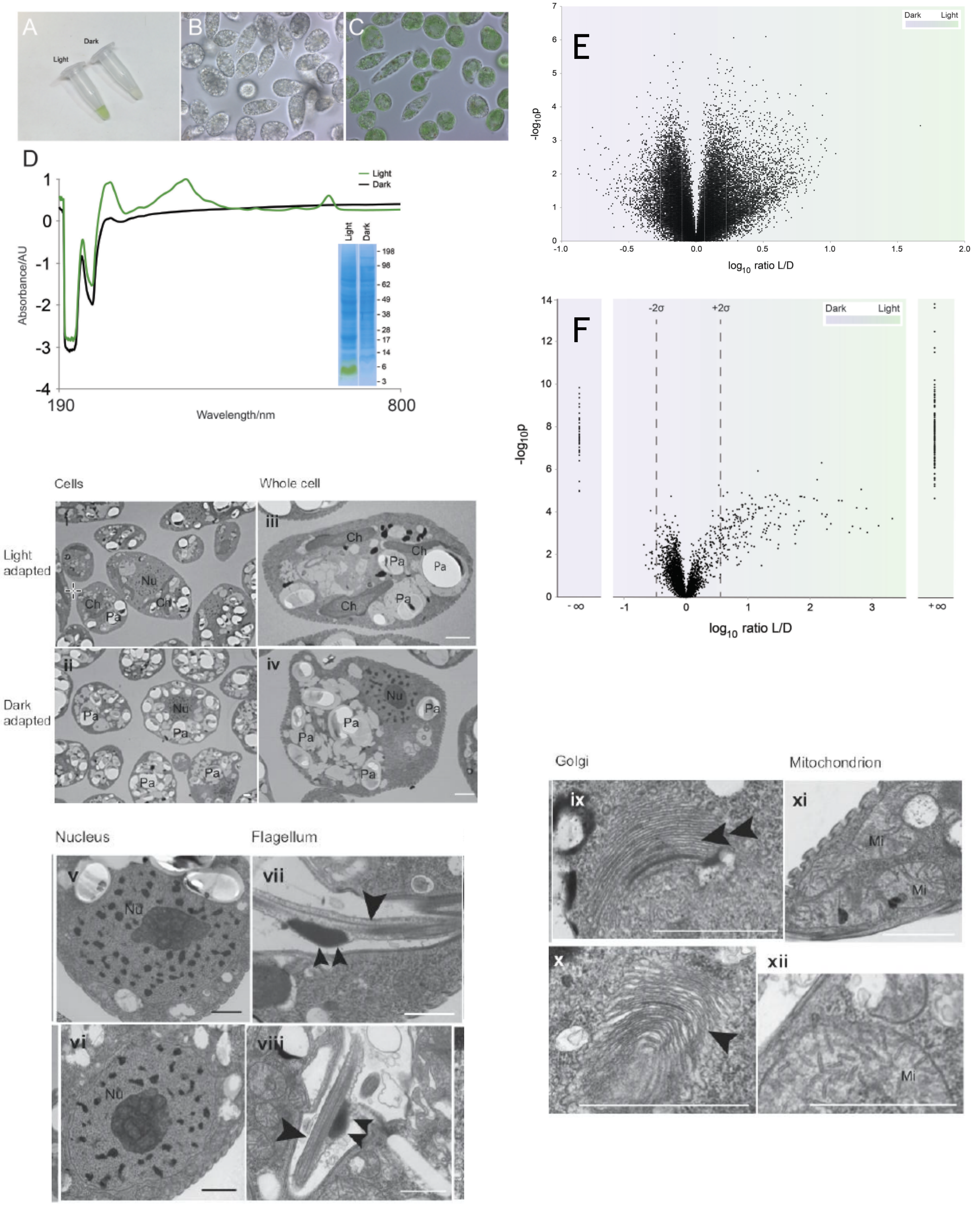

Figure S3: Dark adapted cells lack chlorophyll and have altered proteomes, transcriptomes and ultrastructure.

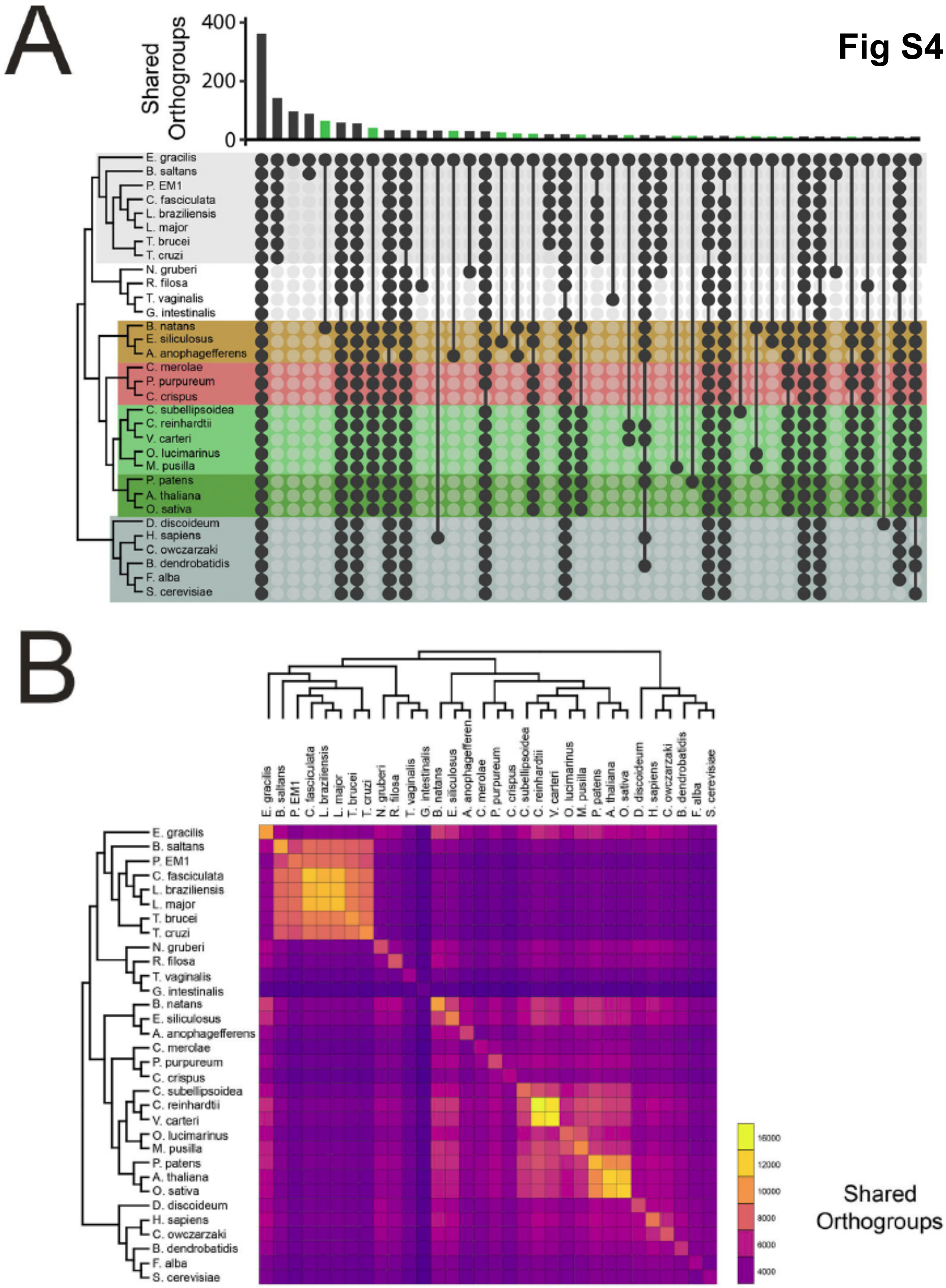

Figure S4: Orthogroup clusters in *E.gracilis* and selected eukaryotes.

Fig S5

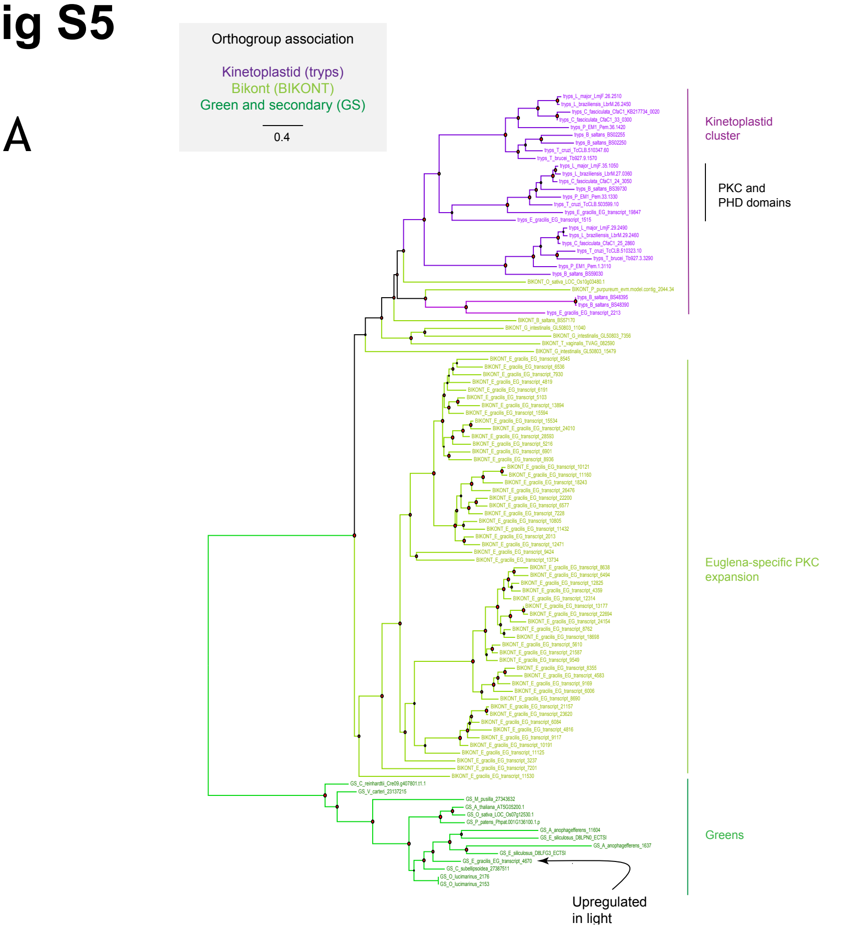

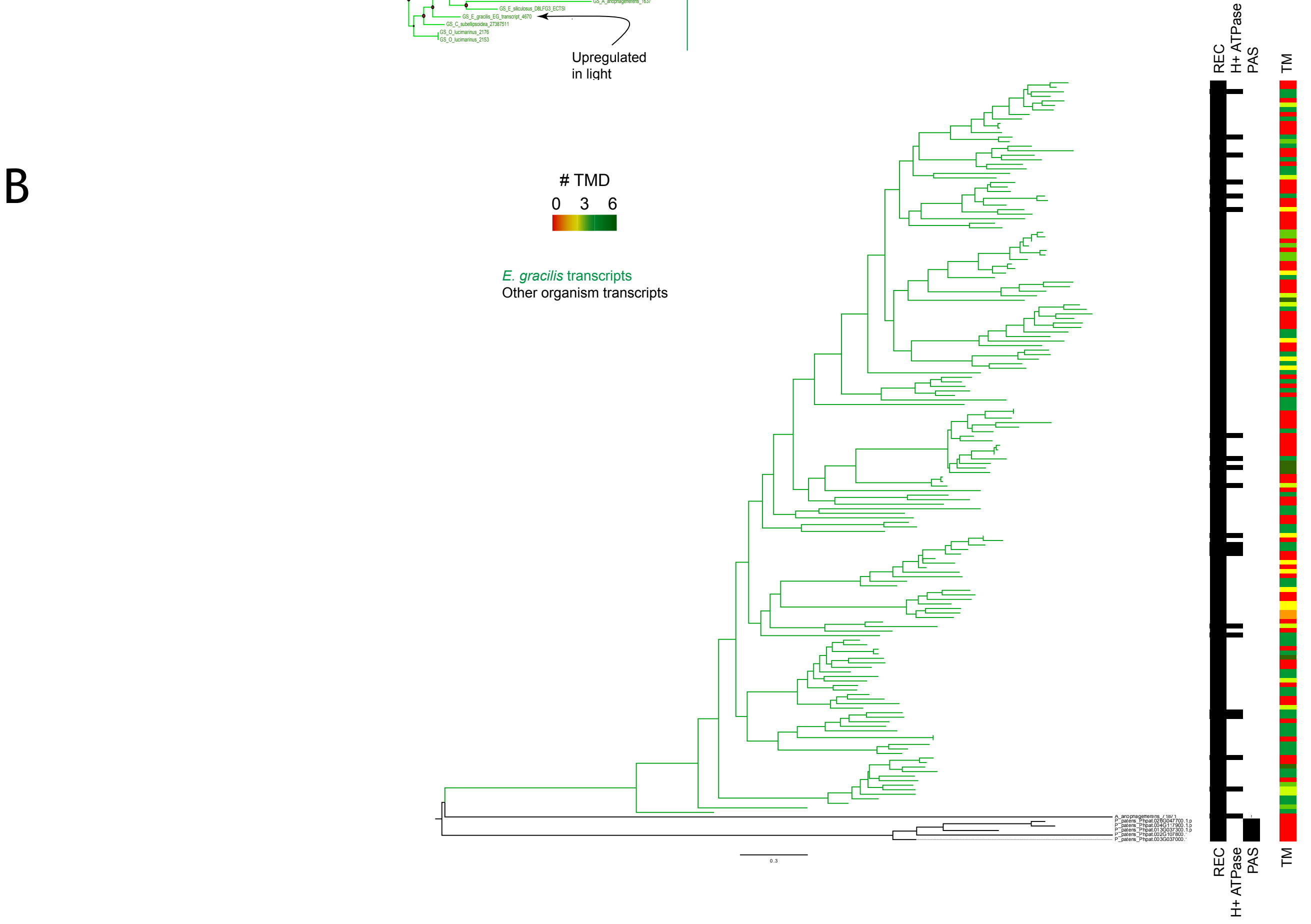

Figure S5: Phylogeny of selected shared large paralog families.

Fig S6A

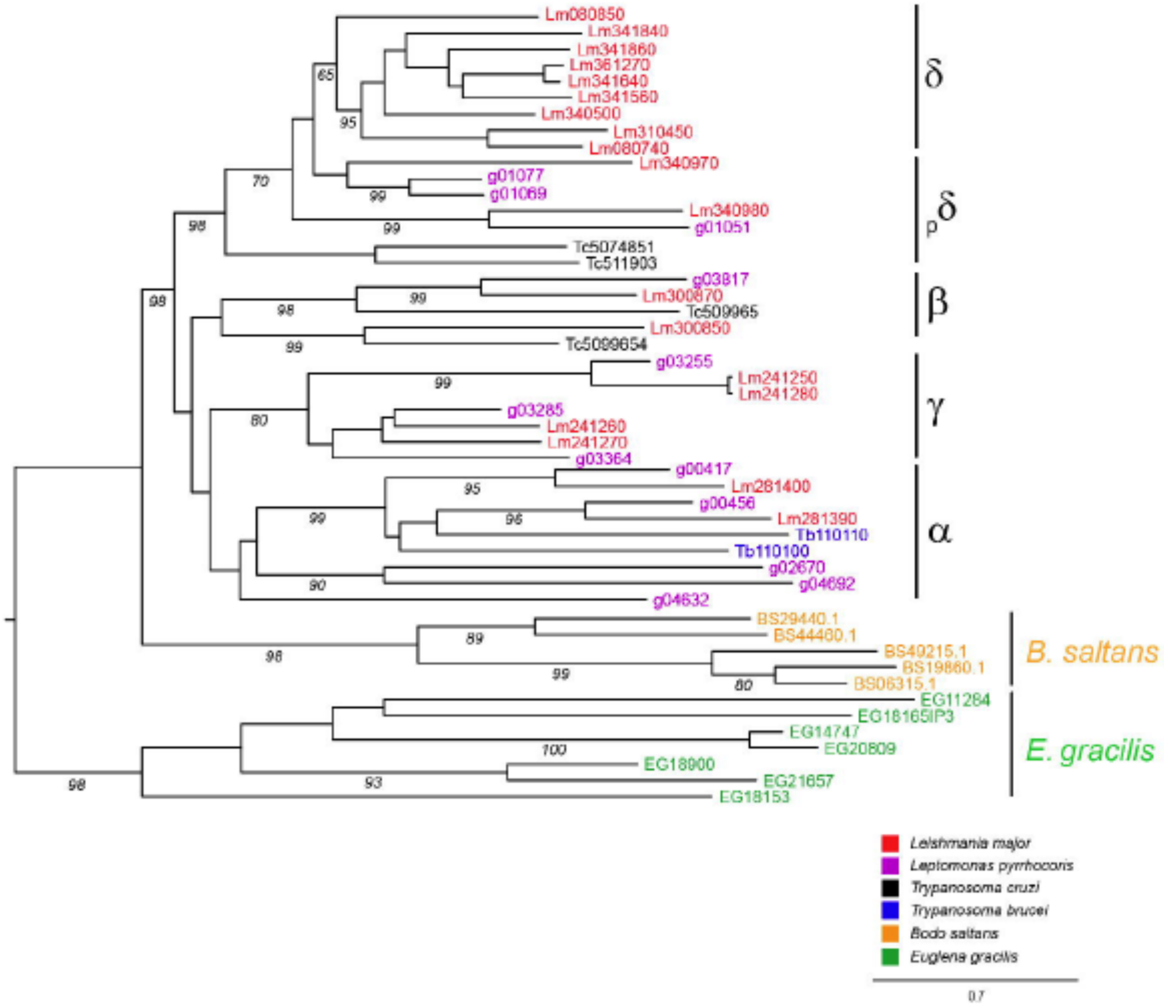

Figure S6: Surface families of *E. gracilis*.

Fig S6BC

B

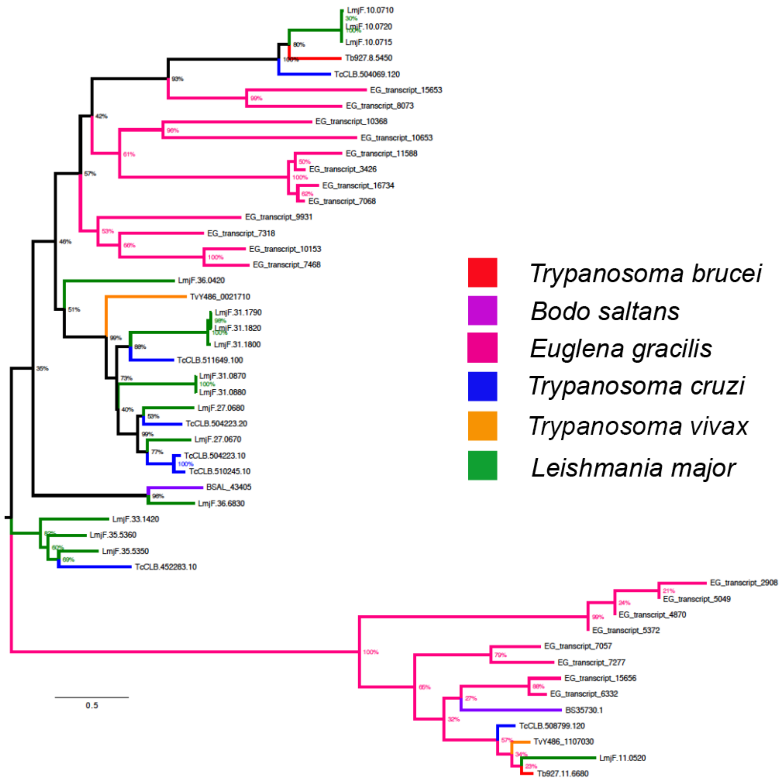

LmjF.10.0710
LmjF.10.0720
LmjF.10.0715
Tb927.8.5450
TcCLB.504069.120
EG_transcript_15653
EG_transcript_8073
EG_transcript_10368
EG_transcript_10653
EG_transcript_11588
EG_transcript_3426
EG_transcript_16734
EG_transcript_7068
EG_transcript_9931
EG_transcript_7318
EG_transcript_10153
EG_transcript_7468
LmjF.36.0420
TvY486_0021710
LmjF.31.1790
LmjF.31.1820
LmjF.31.1800
TcCLB.511649.100
LmjF.31.0870
LmjF.31.0880
LmjF.27.0680
TcCLB.504223.20
LmjF.27.0670
TcCLB.504223.10
TcCLB.510245.10
BSAL_43405
LmjF.36.6830
LmjF.33.1420
LmjF.35.5360
LmjF.35.5350
TcCLB.452283.10
EG_transcript_2908
EG_transcript_5049
EG_transcript_4870
EG_transcript_5372
EG_transcript_7057
EG_transcript_7277
EG_transcript_15656
EG_transcript_6332
BS35730.1
TcCLB.508799.120
TvY486_1107030
LmjF.11.0520
Tb927.11.6680
0.5
Trypanosoma brucei
Bodo saltans
Euglena gracilis
Trypanosoma cruzi
Trypanosoma vivax
Leishmania major

C

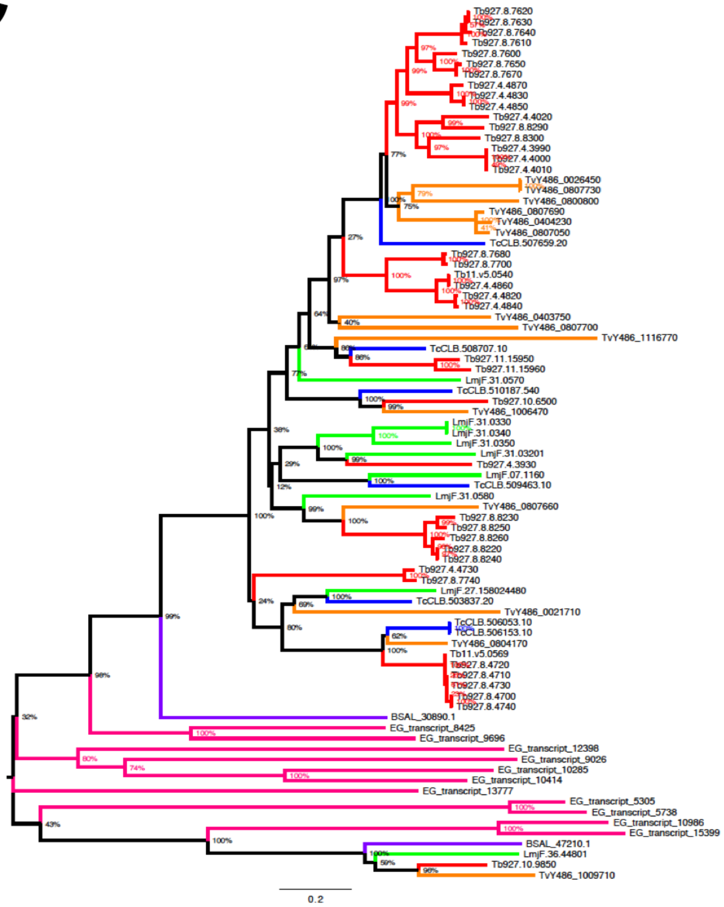

Tb927.8.7620
Tb927.8.7630
Tb927.8.7640
Tb927.8.7610
Tb927.8.7600
Tb927.8.7650
Tb927.8.7670
Tb927.4.4870
Tb927.4.4830
Tb927.4.4850
Tb927.4.4020
Tb927.8.8290
Tb927.8.8300
Tb927.4.3990
Tb927.4.4000
Tb927.4.4010
TvY486_0026450
TvY486_0807730
TvY486_0800800
TvY486_0807690
TvY486_0404230
TvY486_0807050
TcCLB.507659.20
Tb927.8.7680
Tb927.8.7700
Tb11.v5.0540
Tb927.4.4860
Tb927.4.4820
Tb927.4.4840
TvY486_0403750
TvY486_0807700
TvY486_1116770
TcCLB.508707.10
Tb927.11.15950
Tb927.11.15960
LmjF.31.0570
TcCLB.510187.540
Tb927.10.6500
TvY486_1006470
LmjF.31.0330
LmjF.31.0340
LmjF.31.0350
LmjF.31.03201
Tb927.4.3930
LmjF.07.1160
TcCLB.509463.10
LmjF.31.0580
TvY486_0807660
Tb927.8.8230
Tb927.8.8250
Tb927.8.8260
Tb927.8.8220
Tb927.8.8240
Tb927.4.4730
Tb927.8.7740
LmjF.27.158024480
TcCLB.503837.20
TvY486_0021710
TcCLB.506053.10
TcCLB.506153.10
TvY486_0804170
Tb11.v5.0569
Tb927.8.4720
Tb927.8.4710
Tb927.8.4730
Tb927.8.4700
Tb927.8.4740
BSAL_30890.1
EG_transcript_8425
EG_transcript_9696
EG_transcript_12398
EG_transcript_9026
EG_transcript_10285
EG_transcript_10414
EG_transcript_13777
EG_transcript_5305
EG_transcript_5738
EG_transcript_10986
EG_transcript_15399
BSAL_47210.1
LmjF.36.44801
Tb927.10.9850
TvY486_1009710
0.2

Fig S7

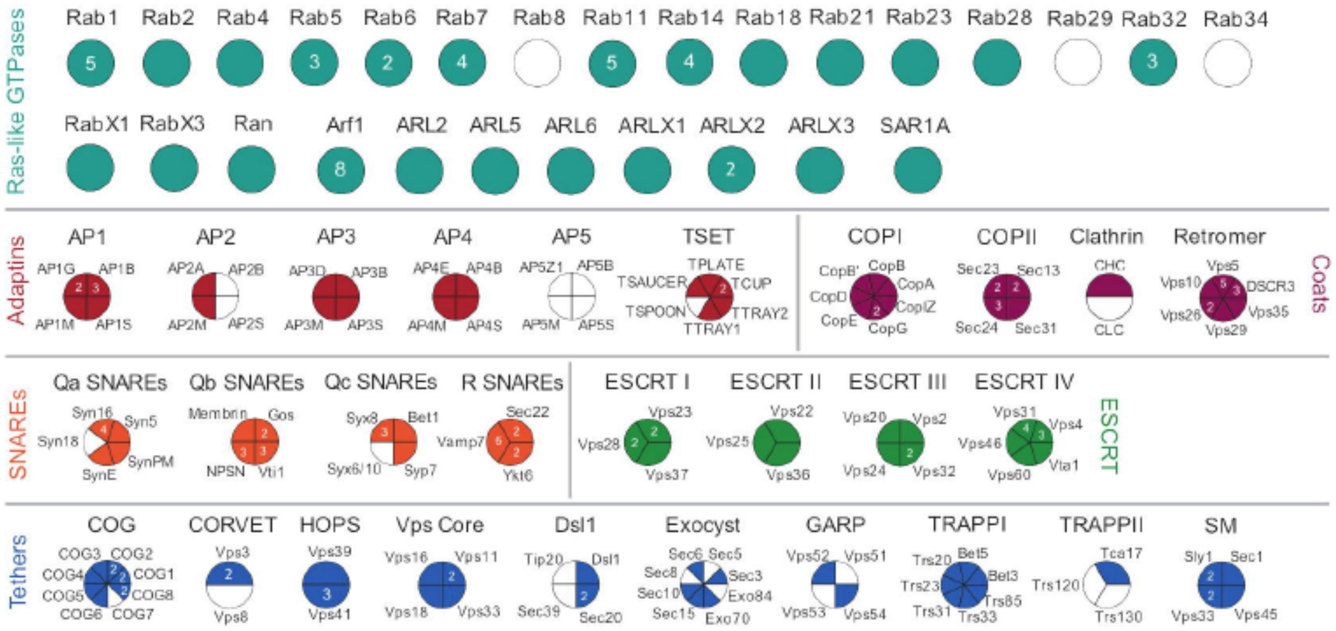

Figure S7: The *E. gracilis* endomembrane system.

Fig S8

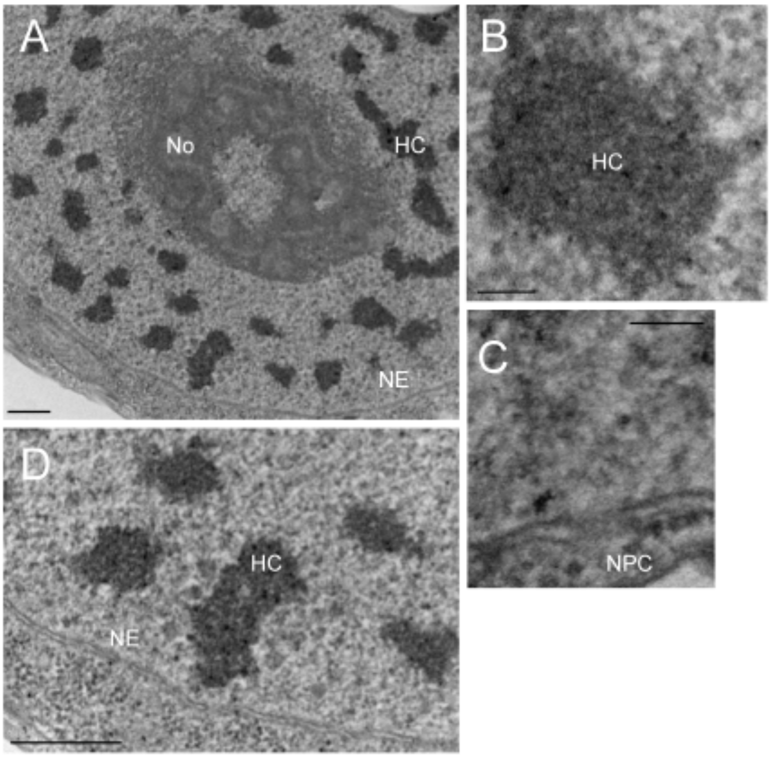

E

Architecture		*T. brucei*	*A. thaliana*	*H. sapiens*	*S. cerevisiae*	*E. gracilis*	Subcomplex
	1	TbNup225	Nup205	Nup205	Nup192	112	
	2	TbNup181	Nup188	Nup188	Nup188	119	Inner ring
	3	TbNup96	Nup93	Nup93	Nic96	2443	
α-solenoid	4*	TbNup158	Nup98	Nup98	Nup145N	2261	
	5	TbNup158	Nup96	Nup96	Nup145C	4195	Outer ring
	6	TbNup89	Nup75	Nup75	Nup85	5081	
	7	TbNup82	Nup107	Nup107	Nup84	2952	
	8	TbNup144	Nup155	Nup155	Nup170	351	Inner ring
	9	TbNup119	Nup155	Nup155	Nup157		
β-propeller α-solenoid (β-α)	10	TbNup152	Nup160	Nup160	Nup120	636	
	11	TbNup132	Nup133	Nup133	Nup133	1761	
	12	TbNup109					
	13	TbSec13	Sec13	Sec13	Sec13	21910	
	14		Seh1	Seh1	Seh1	15911	Outer ring
β-propeller	15	TbNup41	Nup43	Nup43		14251	
	16			Nup37			
	17	TbNup48	ALADIN	ALADIN		0094	
β-propeller, coiled coil RRM, ALPS motif, TM (Trypanosomes)	18	TbNup76	Nup88	Nup88	Nup82	3164	Cytoplasmic (opisthokonts), Symmetric (Trypanosomes)
	19	TbNup65	Nup35	Nup35	Nup53/59	16344	Inner ring
	20		Gp210	Gp210	Pom152	157	
Trans-membrane	21		NDC1	NDC1	NDC1		Pore membrane ring
	22				Pom34		
	23			Pom121			
	24	TbNup110					
	25	TbNup92				2749	Nuclear basket
Coiled Coils	26		NUA	TPR	Mlp1	9861	
	27				Mlp2		

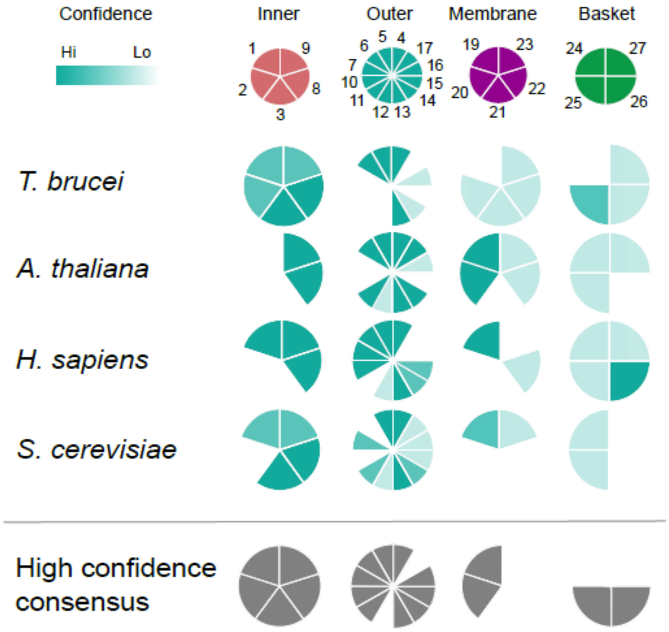

Fig S8

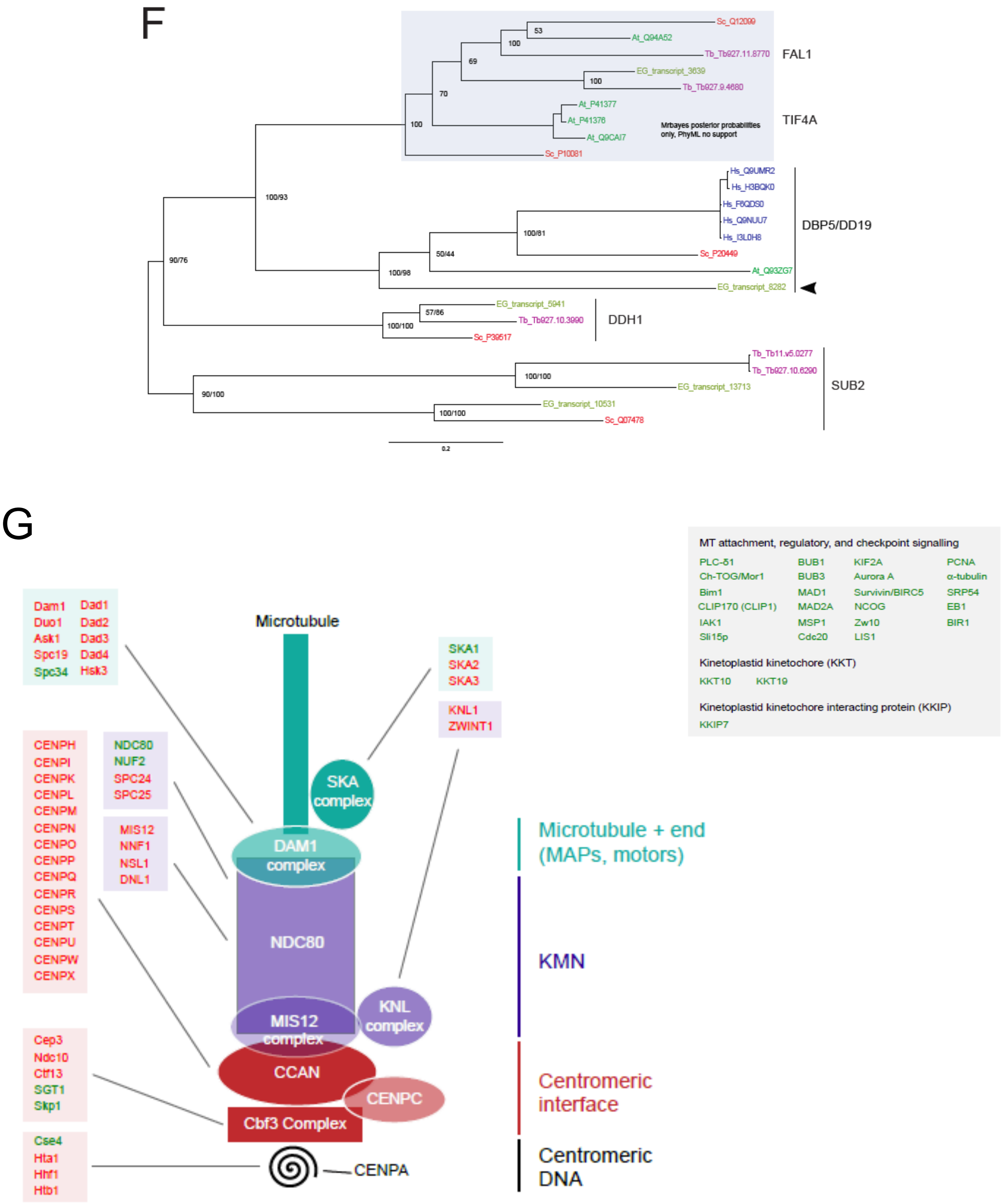

Figure S8: The *E. gracilis* nuclear pore and kinetochore complexes.

Fig S9

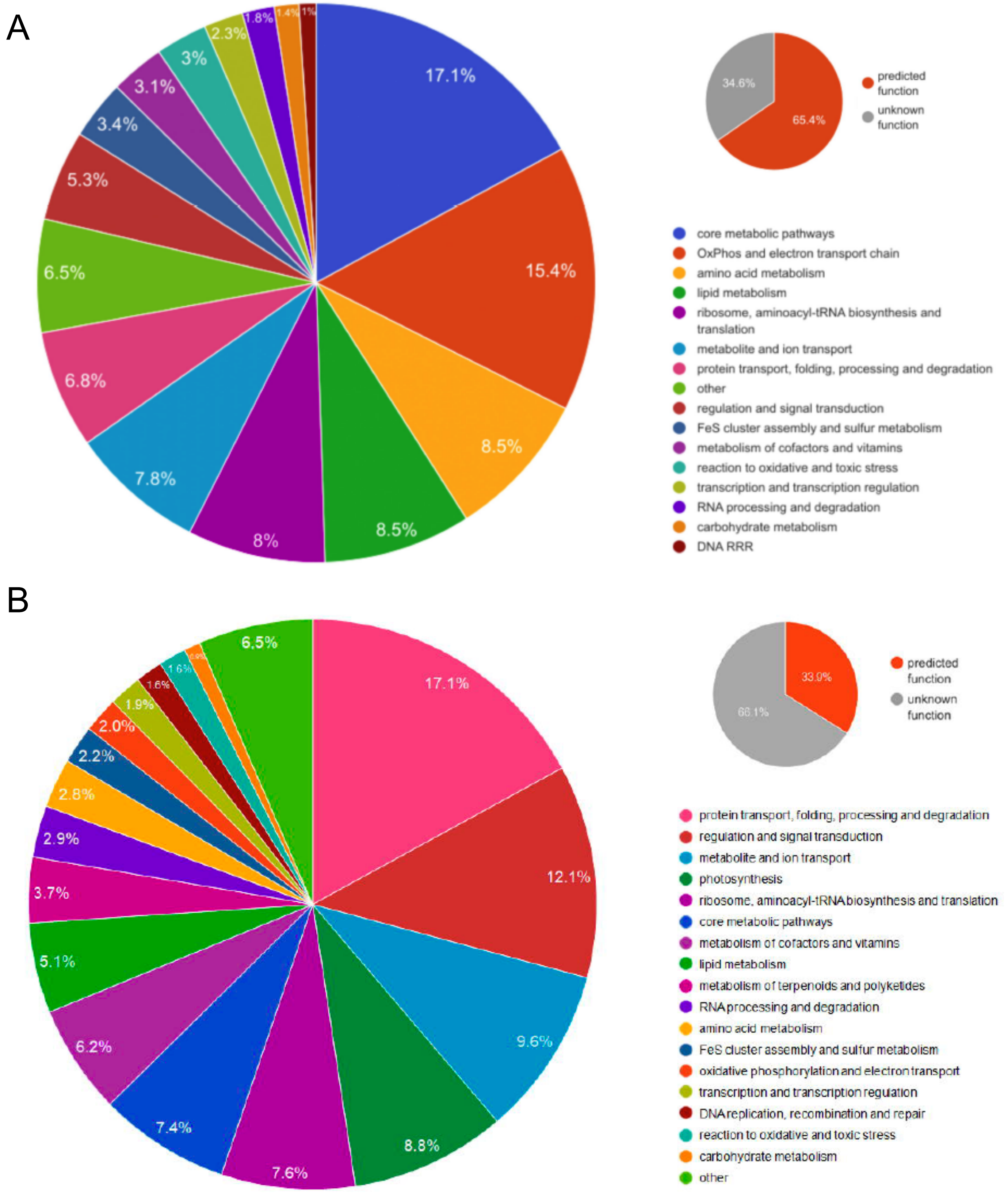

Figure S9: The predicted proteomes of *E. gracilis* organelles.

Fig S10

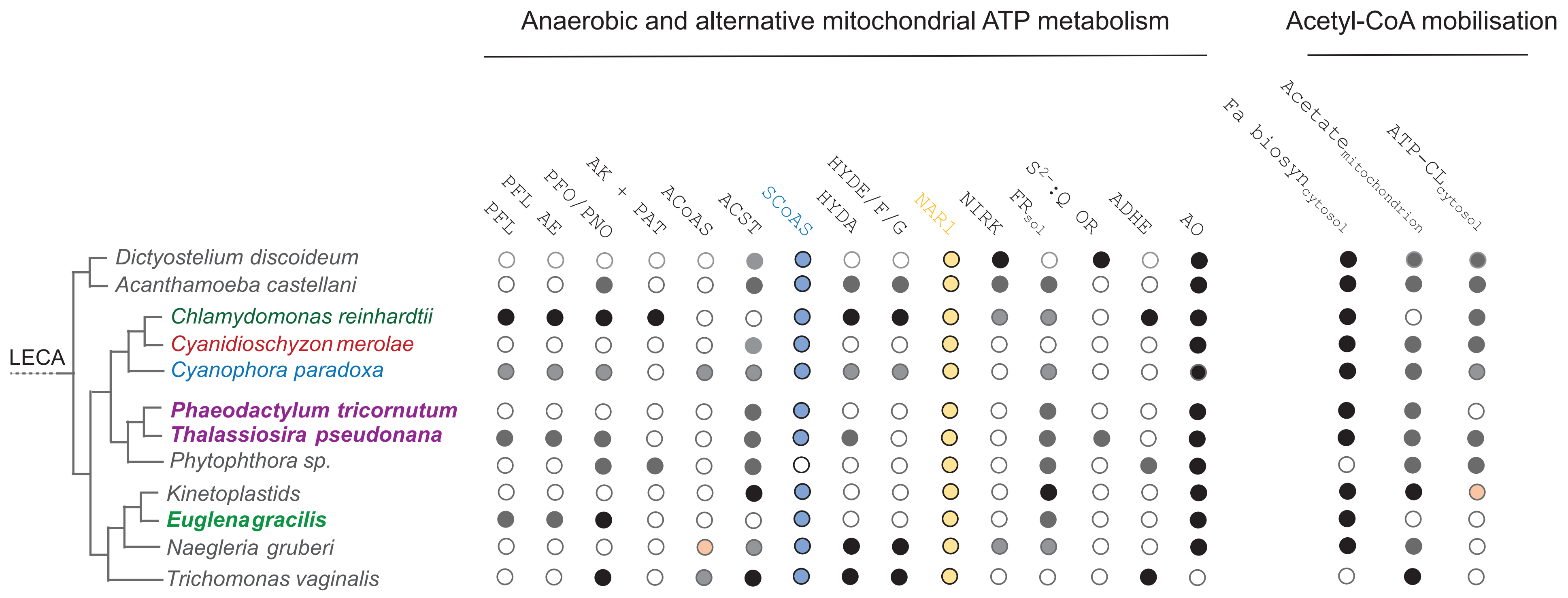

Figure S10: Metabolism in Euglena.

Fig. S11

A

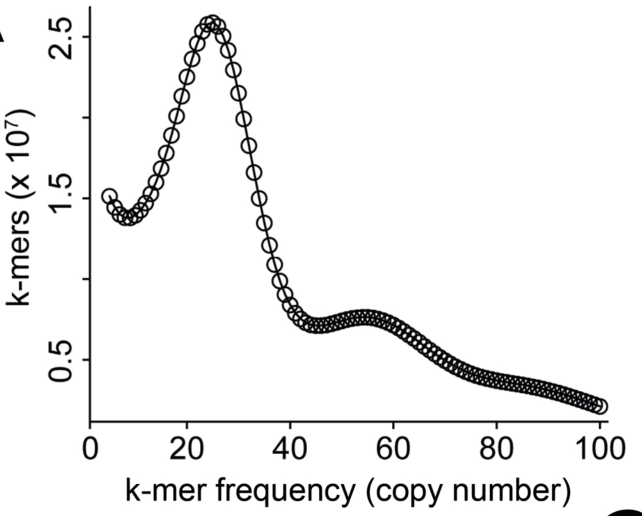

	k-mer size			
	**19**	**21**	**27**	**31**
Heterozygosity (%)	2.53	2.56	2.4	2.19
Haploid Length (mb)	500	443	369	332
Repeat Length (mb)	339	291	230	191
Unique Length (mb)	160	152	139	141

B C

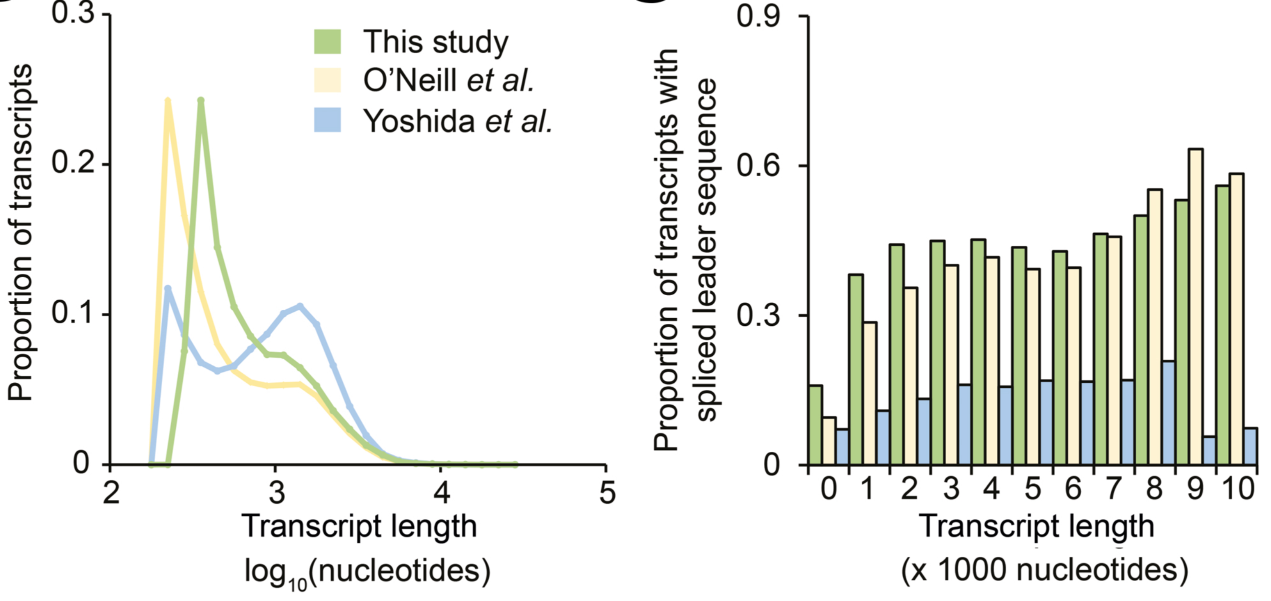

D

**Quality Metric**	**Ebenezer *et al.***	**Yoshida *et al.***
Proportion mapped reads	0.890	**0.960**
Proportion of nucleotides with no coverage	**0.200**	0.350
Proportion of contigs with no coverage	**0.370**	0.430
Proportion of contigs with coverage gaps	**0.890**	0.970
Proportion of contigs with potential chimerism	0.110	**0.060**
Proportion of good contigs	**0.430**	0.400
Transrate score	**0.100**	0.097

Figure S11: Additional assembly features.

Fig. S12

	O'Neill 2015R	O'Neill 2015	Yoshida 2016	Ebenezer 2018	O+Y+E
All unique proteins [authors]	32,128	32,128	not provided	36,526	N/A
All unique proteins [rerun]	40,886	46,650	36,202	36,526	59,678
Complete BUSCOs	259	267	262	255	274
Complete and single-copy BUSCOs	242	240	239	241	240
Complete and duplicated BUSCOs	17	27	23	14	34
Fragmented BUSCOs	11	13	14	23	10
Missing BUSCOs	33	23	27	25	19
Total BUSCO groups searched	303	303	303	303	303
Assembly made by	A. Butenko	O'Neill	Yoshida	Ebenezer	O+Y+E

BUSCO id	O'Neill 2015	Yoshida 2016	Ebenezer 2018	Concatenation	Annotation
EOG093704J7	absent	present	present	present	DNA helicase
EOG09370CK6	absent	absent	absent	absent	General transcription factor IIE 56 kDa subunit
EOG09370CTU	absent	absent	absent	absent	Protein BING1
EOG09370E36	absent	absent	absent	absent	Histone acetyltransferase 1
EOG09370FXE	present	present	absent	present	Surfeit 1
EOG09370GEO	absent	absent	absent	absent	Origin recognition complex, subunit 2
EOG09370HVI	present	absent	present	present	Lipoate biosynthesis protein
EOG09370I7R	absent	absent	absent	absent	ATP-binding domain-containing protein 3
EOG09370KWF	absent	absent	absent	absent	COS16 homolog
EOG09370NBW	absent	absent	absent	absent	IWS1-like protein
EOG09370O51	present	absent	present	present	Electron transfer flavoprotein subunit beta
EOG09370OS5	absent	absent	absent	absent	28S ribosomal protein S2, mitochondrial
EOG09370P2W	present	present	absent	present	Signal recognition particle 19 kDa protein
EOG09370P7I	absent	absent	absent	absent	ATP12 homolog
EOG09370QCX	absent	absent	absent	absent	Translocation protein 1
EOG09370QT3	absent	absent	present	present	Ribonuclease P protein subunit p29
EOG09370XFG	absent	absent	absent	absent	GINS complex subunit 3
EOG09370Y63	absent	absent	present	present	40S ribosomal protein S13
EOG09370YC4	absent	absent	absent	absent	Nuclear receptor subfamily 2 group B member 2
EOG0937106Y	absent	absent	present	present	Density-regulated protein
EOG093710A7	present	absent	absent	present	MNAT CDK-activating kinase assembly factor 1

EOG093711QY	present	present	absent	present	tRNA methyltransferase 11-2 homolog
EOG0937122Q	absent	absent	absent	absent	TAF13 RNA polymerase II, TATA box binding protein (TBP)-associated factor, 18kDa
EOG0937128O	present	present	absent	present	G antigen 2A
EOG0937129K	absent	absent	absent	absent	Defender against cell death 1
EOG093712G8	absent	absent	absent	absent	Complex I-19kD
EOG093712Q6	present	absent	present	present	RNA polymerase II (DNA directed) polypeptide F
EOG09371431	present	absent	present	present	40S ribosomal protein S20
EOG093714JU	absent	absent	absent	absent	Ubiquitin-related modifier 1
EOG093717LU	absent	absent	absent	absent	DNA-directed RNA polymerase III subunit L
EOG093718E9	absent	absent	absent	absent	Complex I-B8
EOG093718EG	absent	absent	absent	absent	Mitochondrial import inner membrane translocase subunit Tim10
EOG093719M8	present	present	absent	present	Tubulin folding cofactor A
Total undetected	23	27	25	19	

Figure S12: BUSCO analysis and comparison of E. gracilis transcriptomes

Additional file 1.pdf: Supplementary figures

Figure S1: Organisation of open reading frames in the *E. gracilis* genome. Predicted exons are shown for contigs, where transcript data are mapped. Black lines indicate the mapped contig span, and yellow rounded rectangles indicate exons. Note that there are highly variable structures present, with several ORFs predicted as lacking introns, whilst others are highly fragmented. Transcript numbers mapping to contigs are shown at left, and the contig length is indicated by a scale bar (in kb). Note also that in a number of cases several transcripts map to a given contig, for example contig 717355 maps against six transcripts (239, 254, 260, 297, 495 and 579).

Figure S2: Functional analysis of Euglena coding capacity by Gene Ontology. The orthogroup clusters identified in the *E. gracilis* predicted ORFs, grouped similarly as Figure 3, were analysed for GO annotation using $Blast_2GO$ and Interpro. The first GO term given for each main category (biological process, molecular function, or cellular component), was converted to *GO generic slim*. Panel A: Number of protein groups with GO annotation in each cluster shown as green (annotation retrieved) or blue (no annotation). Panel B: GO annotation for each cluster. Each GO term had to be represented by > 5% of the total annotated genes in each cluster for inclusion. Histograms are normalised to the total number of ORFs per cluster.

Figure S3: Dark adapted cells have altered proteomes and transcriptomes. All panels represent cells following subculturing and six days in the light or dark. Panel A: loss of pigments from dark cultured cells. Panels B and C: Phase contrast images of cells after dark and light culture respectively. Panel D: UV-VIS absorbance spectra demonstrating loss of absorbance associated with chlorophyll and other pigments. Inset: Coumassie-stained 1D SDS-PAGE. Left lane, light and right, dark cultured cells. Molecular weight standards (kDa) at right. Panel E: Volcano plot of RNAseq data for dark against light grown cells. Data are the mean of triplicate RNA extractions. Panel F: Volcano plot of mass spectrometric proteomic analysis for dark against light grown cells. Data from triplicate analyses. Dotted lines indicate significance threshold for altered abundance, and groups at far left and far right are proteins detected in only one condition, i.e. infinite change. Panel G: Transmission electron micrographs comparing ultrastructure of cells from dark and light adapted culturecultures. Both light and dark-adapted cells contain paramylon granules (Pa) (i,ii) but more extensive in dark-adapted cells (ii). Light-adapted cells contained

multiple mature chloroplasts (Ch) (iii) absent from dark-adapted cells (iv). Both light- and dark-adapted cells contained nuclei with prominent nucleoli and many smaller electron dense foci in surrounding nucleoplasm (Nu) (v,vi). Both light- and dark-adapted cells contained flagella (arrow) with associated paraflagellar body (double arrow) (vii,viii). Both light- and dark-adapted cells contained Golgi apparatus with numerous narrow cisterna (arrow) (ix,x). Both light- and dark-adapted cells contained mitochondria with narrow cristae (Mi) (xi,xii). Scale bar in all panels ~1μm.

Figure S4: Orthogroup clusters in *E. gracilis* and selected eukaryotes. Panel A: Orthogroups shared between *E. gracilis* and additional taxa are shown, using an identical taxon dataset as in Figure 3, but grouped into higher order taxa for finer resolution. The top shows the number of shared orthogroups within each cluster, with clusters shared between Euglena and other photosynthetic taxa highlighted in green. The main graphic grid indicates the taxa that share an orthogroup with Euglena by the presence of a filled dot and a tie bar; for example the leftmost orthogroup is pan-eukaryotic, whilst second left is restricted to the euglenids. A schematic phylogenetic tree is shown at far left indicting the phylogenetic relationships between the taxa. Euglenids are shown in gray, other excavates in white, brown algae in brown, red algae in red, unicellular plants in light green, and vascular plants in dark green. Bikonts (Amoebozoa plus opisthokonts) are in dark gray. Panel B: Representation of orthogroups shared between all taxa analysed in a pairwise manner plotted as a heat map. Lighter colours (yellow) indicate higher numbers of shared orthogroups whilst darker (purple) indicate fewer shared orthogroups. Phylogenetic relationships are shown above and to the left of the species names.

Figure S5: Phylogeny of selected shared large paralog families. Maximum likelihood trees and domain annotations for PKC (Panel A) and Rec domain-containing (Panel B) gene families extracted from the orthogroup cluster analysis that are shared between *E. gracilis* and additional taxa (OG000113 and OG000137). Specific lineages and orthogroup associations features are colour-coded as indicated in the key. Note the presence of hugely expanded gene families specific for *E. gracilis* in each case. In panel B, the number of *trans*-membrane domains (TMD) is indicated by a heat map, and the presence of REC, ATPase and PAS domains by a black bar. Annotation of transcript numbers gave been omitted from panel B for clarity. For an additional example see Figure 4.

Figure S6: Surface families of *E. gracilis*. Phylogenies for amastin (panel A), amino acid permease (panel B) and amino acid transferase (panel C) gene families extracted from predicted surface families. Phylogenies were reconstructed using neighbour joining and protein sequences from *Euglena* and diverse kinetoplastids. The phylogeny was estimated with MEGA6 using a neighbour-joining method with a JTT model and 100 bootstrap replicates. Terminal nodes are named with Genedb (http://www.genedb.org/Homepage/ http://www.genedb.org/Homepage/) and Tritrypdb (http://tritrypdb.org/tritrypdb/http:// tritrypdb.org/tritrypdb/) identifiers, internal nodes are labelled with bootstrap percentages, branches are shaded according to species as indicated. Trees are mid-point rooted.

Figure S7: The *E. gracilis* endomembrane system. Membrane trafficking proteins were retrieved using BLAST and HMMer searches with query sequence from reference taxa. Ortholog identity and paralog numbers were confirmed using phylogenetics of individually gene families. Filled circles represent the presence of the protein and white/open represents candidate predicted proteins that were not retrieved by search methods. Paralog numbers are shown in white for, from the top, Rab, Arf, ARL, and Sar GTPase protein families, adaptin and coatomer protein complexes, SNARE proteins, endosomal protein complexes and multi-subunit tethering complexes.

Figure S8: The *E. gracilis* nuclear pore and kinetochore complexes. Panel A - D: Unusual 'currant bun' arrangement of heterochromatin in the interphase nucleus of *E. gracilis*. NE; nuclear envelope, HC; heterochromatin, No; nucleolus and NPC; nuclear pore complex. Scale 500nm Panel A/C, 100nm Panel B/D. Panel E: Top predicted orthology relationships for *E. gracilis* nucleoporins compared to human, yeast, plant and African trypanosomes. NuclearNucleoporins are grouped according to secondary structure (left) and subcomplex (right) and colour coded; inner ring red, outer ring blue, pore membrane and nuclear basket in pure and green respectively. Orthologs for reference species and predicted *E. gracilis* transcripttranscripts are shown. Open cells indicate absence of *E. gracilis* candidate and empty coloured cells where reference species lacks ortholog. lowerLower panel Coulson plot for subcomplexes using numerical scheme in table. Colours indicate confidence with which Euglena orthologs are ascribed, white is no hit. psi-BLAST threshold of $1e^{-4}$ was used. Panel F: Phylogenetic evidence for a Euglenid Dbp5 RNA helicase. A maximum-likelihood/MrBayes phylogenetic reconstruction of Dbp5 orthologs and their closest relatives is shown. *E. gracilis* has a clear Dbp5 ortholog (arrowhead). *S. cerevisiae*; red, *A. thaliana*; green, *E. gracilis*; khaki, *T. brucei*; purple, *H. sapiens*: blue. Panel G: Reconstruction of *E. gracilis* kinetochore. Individual subcomplexes

are coloured teal for MT proximal, red for DNA-proximal and blue of the KMN complex and highlighting the microtubule plus-end (Dam1 and SKA1), the KMN (NDC80, MIS12 complex, KNL1 complex), the centromeric interface (CCAN, CENPC, Cbf3 complex), and centromeric DNA (CENPA). Canonical subunits coloured red for unidentified and green for present in *E. gracilis*. KKTs and KKIPs are restricted to the kinetoplastids but some subfamilies (KKT10/19 and KKIP7 respectively) are present in *E. gracilis*.

Figure S9: The predicted proteomes of *E. gracilis* organelles. Panel A: Mitochondrion. Proteins predicted to be targeted to the mitochondrion and encoded by the nuclear genome were identified as described in methods. Inset: The ratio between annotated proteins with a certain predicted function and proteins of unknown homology and/or function. In the set of 1075 mitochondrial candidates, 703 (65.4%) remained with a predicted function while 372 (34.6%) do not possess a predicted function. Main pie graph: Proteins with predicted functions were used for metabolic and cellular pathways reconstruction and sorted into 16 functional categories. The reconstruction of likely metabolic pathways present within the organelle are shown in Figure 5. Panel B: Plastid. Proteins predicted to be targeted to the plastid and encoded by the nuclear genome were identified as described in methods. Inset: The ratio between annotated proteins with a certain predicted function and proteins of unknown homology and/or function. In the set of 1,902 plastid candidates, 1,257 proteins (66.1%) remained without a predicted function, 1,008 being completely unidentifiable with no homologs in bioinformatic databases and 249 having unclear or completely unknown function. Main pie graph: The remaining 645 (33.9%) proteins with predicted function were used for metabolic and cellular pathways reconstruction and sorted into 18 functional categories. The largest portion of proteins were those involved in the synthesis, post-translational modification and folding of newly synthesized proteins and with regulatory and/or signaling functions. Other major categories include photosynthesis and chlorophyll biosynthesis, ribosome biogenesis and protein synthesis and transport of ions and non-protein molecules. A reconstruction of likely metabolic pathways present within the organelle are shown in Figure 6.

Figure S10: Metabolism in *E. gracilis.* Left: Anaerobic and alternative mitochondrial ATP metabolism. For comparisons with *E. gracilis*, protists were chosen based on predicted or known metabolic flexibility (Ac, Cr, Cp, Tp, P sp, Ng), obligate anaerobic metabolism (Tv), little known anaerobic metabolic potential (Dd, Cm, Pt) or evolutionary closeness to *Euglena* (kinetoplastids). Black, enzyme(s) biochemical characterized; grey, candidate

orthologue present; orange, an enzyme no longer predicted to function as a candidate ACoAS in *N. gruberi*; blue, SCoAS functions in both ATP-dependent acetate production (in a cycle with ACST) and as an enzyme of the Krebs cycle; yellow, candidate Nar1 protein conserved in all eukaryotes and a component of eukaryotic cytosolic Fe-S cluster assembly; open, no ortholog/homolog detected. Left; Acetyl-CoA mobilisation: FA biosyn$_{cytosol}$, black circles indicate a requirement for cytosolic fatty acid biosynthesis and open circles indicate no capacity for fatty acid biosynthesis is known; Acetate$_{mitochondrion}$, black circles indicate experimentally determined production of acetate in the mitochondrion (or hydrogenosomes in the example of *Trichomonas vaginalis*), grey circles indicate a prediction for mitochondrial acetate production, and open circles indicate no obvious predicted capacity for mitochondrial acetate production; ATP-CL$_{cytosolic}$, grey circles indicate the prediction ATP-dependent citrate lyase, the enzyme that yields acetyl-CoA from mitochondrially exported citrate and open circles indicate no ATP-dependent citrate lyase detected.

Figure S11: Additional assembly features. Panel A: k-mer spectral plot generated from the combined Illumina read data. The spectrum indicates an estimated 25x coverage of the single copy component of the genome with a long tail that represents the repeats. Right shows variance of calculated assembly size depending on the assumed k-mer size. Panel B: Lengths of transcripts in the assembly described here and O'Neill et al., (17) and Yoshida et al., (19). Panel C: Comparison of transcript size and proportion of transcripts with spliced leader sequence at 5' the end for the present work and that from O'Neill et al., and Yoshida et al., (17, 19). Panel D: Contig assembly and quality metric comparisons between the present dataset and (19).

Figure S12: BUSCO comparisons between the present work and prior transcriptomes. Top panel: Except O'Neill2015R, all assemblies were made by the authors. O'Neill2015R was an independent reassembly by A. Butenko, and data provided with thanks. All other assemblies was analyzed in the exactly same way (transdecoder+cdhit then busco). O+Y+E was made by concatenation of all three sets of proteins and than cd-hit to remove redundancy. Published numbers of proteins and those from this analysis are in rows 2 and 3. Lower panel: Unfound BUSCOs from all three datasets. Note that here is considerable concordance between the BUSCOs that could not be identified and which suggests similar data quality as well as probable near saturation in terms of coverage that is possible with current sequencing and assembly approaches.
