# Supplementary material for: Transcriptome, proteome and draft genome of Euglena gracilis
Source: BMC Biol. 2019 Feb 7;17:11. doi: 10.1186/s12915-019-0626-8 (PMC6366073; doi:10.1186/s12915-019-0626-8)
Supplement: Supplementary file 6 — Supplementary analyses. (DOCX 17 kb) [file 12915_2019_626_MOESM6_ESM.docx]

A: Statistics of predictions for signal peptide, *trans*-membrane domains and GPI anchors.

|  | Proportion of total transcripts with signal peptide |  | Of which contain a signal peptide and trans-membrane domain | Of which contain a signal peptide and GPI signal | Of which contain a signal peptide only |
| --- | --- | --- | --- | --- | --- |
| Number of putative proteins | 3259(9%) |  | 1253(38%) | 299(9%) | 1707(52%) |
| Conserved (with BLAST hit) | 1622(50%) |  | 672(53%) | 187(63%) | 763(45%) |
| Conserved (no BLAST hit but with orthogroup) | 939 (29%) |  | 356(28%) | 70(23%) | 513(30%) |
| Euglena-specific | 698(21%) |  | 225(18%) | 42(14%) | 431(25%) |

B: Frequency distribution of predicted *Euglena*-specific surface gene families.


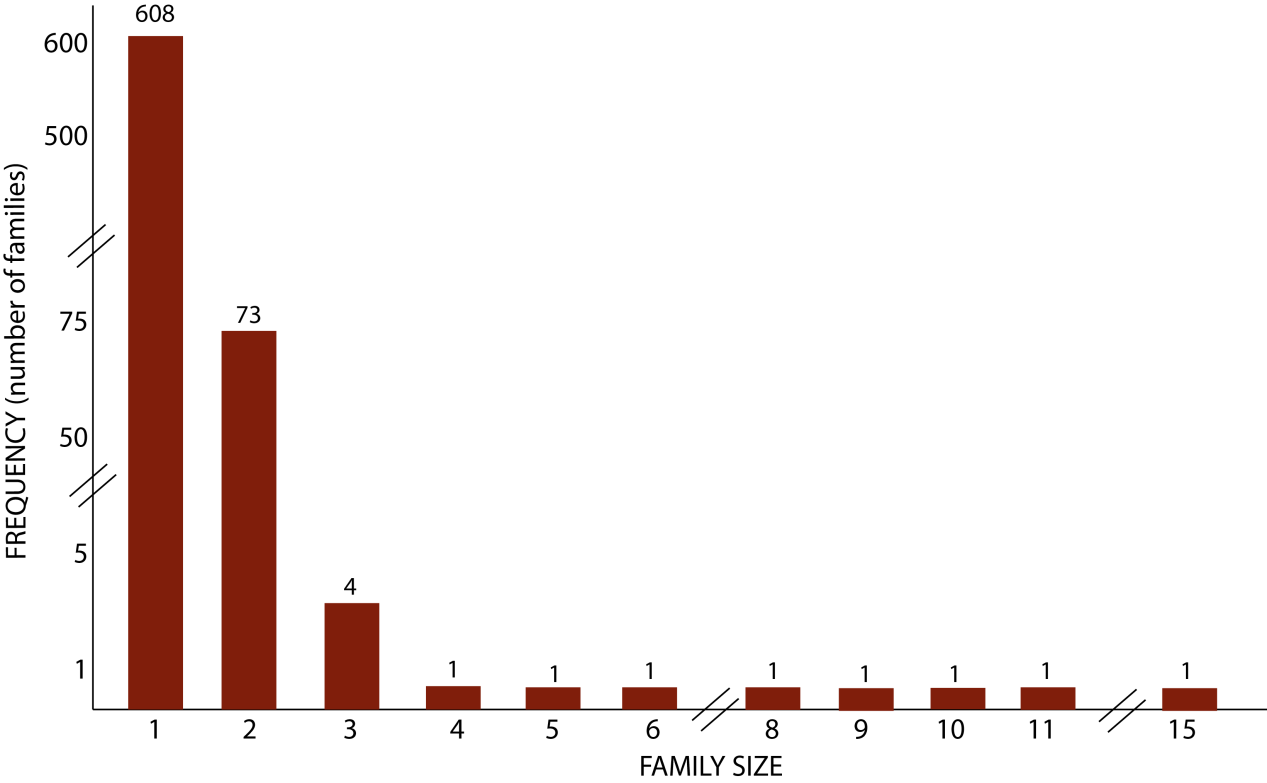


C: PHYRE 2.0 summary results for an element of each multi-copy family (n>4) of *E. gracilis*, including family size, residues matching the model and correspondent coverage of the sequence, percentage identity, confidence of prediction, and description of top template model.

| Transcript | Size | Residue coverage(%) | ID% | Confidence | Top template |
| --- | --- | --- | --- | --- | --- |
| EG_transcript_11397 | 5 | 94(19%) | 13% | 77% | signaling protein |
| EG_transcript_12171 | 9 | 16 (4%) | 31% | 38.40% | Hairpin loop containing domain-like |
| EG_transcript_1911 | 8 | 192(32%) | 12% | 97.30% | transferase/transferase inhibitor |
| EG_transcript_19669 | 5 | 44(13%) | 32% | 42.70% | DNA methylase specificity domain |
| EG_transcript_226 | 6 | 38(8%) | 21% | 41.90% | membrane protein |
| EG_transcript_2946 | 15 | 215(39%) | 13% | 100% | transferase |
| EG_transcript_3761 | 11 | 215(39%) | 20% | 94.40% | transferase |
| EG_transcript_4440 | 10 | 116(15%) | 15% | 97% | transferase |
