# Supplementary material for: Transcriptome, proteome and draft genome of Euglena gracilis
Source: BMC Biol. 2019 Feb 7;17:11. doi: 10.1186/s12915-019-0626-8 (PMC6366073; doi:10.1186/s12915-019-0626-8)
Supplement: Supplementary file 8 — Table S6. Predicted proteomes for the E. gracilis plastid and the mitochondrion. Panels include summaries for each organelle for numbers of genes in functional categories found, annotations for transcripts predicted as mitochondrial or chloroplastic and finally a reconstruction of major mitochondrial complexes and pathways. (DOCX 141 kb) [file 12915_2019_626_MOESM8_ESM.docx]

**Supplementary analysis**

***Meiosis:*** A panel of 25 meiotic genes from ciliates were used to identify possible orthologs, and identified 16 candidates (Supplementary file 5, Table S4). Seven of these genes are conserved in at least one ciliate. Of these seven conserved genes, six were found and classified into functions in *E. gracilis* suggesting the presence of conserved meiotic activities. REC8 is the only conserved meiotic specific gene we could not find. The absence of this gene may be due to the incomplete dataset or that *E. gracilis* may have a different way of regulating sister chromatid cohesion and recombination between homologous chromosomes (Parisi, *et al.,* 1999). Overall, multiple copies of HOP2 (meiosis-specific) and meiosis related (RAD23, RAD50, KU70, LIG4/DNL, EXO1, and MSH2) genes were identified in the *E. gracilis* transcriptome suggesting expansions in copy numbers. Multiple copies of meiotic genes have also recently been associated with parthenogenesis, asexual reproduction, which is present in major lineages (Schurko, et al., 2015).

***Bilobe and associated proteins***: In kinetoplastids, the bilobe plays a central role in Golgi, flagellar pocket collar and flagellum attachment zone biogenesis (Zhou, *et al.*, 2010). Five (MORN1, RRP1, BILBO1, Centrin-2 and Centrin-4) of the six proteins which localizes within the bilobe structure - a discrete cytoskeletal structure and tightly associated with flagellar skeleton (Esson, *et al.,* 2012; Morriswood, *et al.,* 2013; Wang, *et al.,* 2012; Zhou, *et al.,* 2010; McAllaster, *et al.,* 2015; Aslett, *et al.,* 2010; Bugreev, *et al.,* 2011) - were found in *E. gracilis* in multiple copies*.* PLK proteins were missing. Centrins were integral to the discovery of the bilobe, as it was first observed using the pan-centrin monoclonal antibody 20H5 (Esson, *et al.,* 2012). The bilobe architecture in *E. gracilis* is predicted as similar to that found in trypanosomatids and possibly performs similar functions which include precise positioning, biogenesis, and inheritance of single-copied structures of the Golgi apparatus during cell cycle (Gheiratmand and He, 2014). This is significant because the bilobe structure and associated proteins play a role in facilitating protein entry into the cell, thus providing a link between the cytoskeleton and the endomembrane system (Morriswood et al., 2015), bilobe and FAZ biogenesis, consequently affecting flagellum-driven cell motility and division (Brasseur et al., 2014).

***Signal transduction:*** Signal transduction genes were identified in *E. gracilis* using BLAST and Blast_2_GO. Supplementary file 5, Table S4 documents the distribution of predicted signal transduction genes. Sequences showing similarity to protein kinase, followed by cyclase (including both adenylate and guanylate cyclases), heat-shock protein, flagellar protein, photoactivated adenylate cyclase, kinesin, histidine kinase, histone, calmodulin, inositol 1,4,5-trisphosphate receptor and mechanosensitive channels were noted. The presence of these large number of signal transduction and transporter sequences suggests that *E. gracilis* has a remarkable richness of channel regulation for signal transduction in response to diverse stimuli, which ensures its survival and adaptation to a wide array of habitats it colonises.

***Histones*:** Five major families of histones exist: H1/H5, H2A, H2B, H3, and H4 (Cox, *et al.,* 2005; Bhasin, *et al.,* 2006; Hartl, *et al.,* 1988). Histones H2A, H2B, H3 and H4 are known as the core histones, while histones H1 and H5 are known as the linker histones (Tilber, *et al.,* 2012). In the *E. gracilis* transcriptome, we found all histone variants (Additional file 5, Table S4, Talbert, *et al.,* 2012).

***Translational apparatus*:** *T. brucei* translational apparatus reference sets (Aslett, *et al.,* 2010) were used to predict the minimal translational apparatus in *E. gracilis.* Overall, 158 translational genes were found in *E. gracilis* and include ribosomal proteins (70), aminoacyl-tRNA synthases (50), translational initiation factors (18), elongation factors (6) and termination factors (3) (Grosjean, *et al.,* 2014; Hernández, 2012; Hernández, *et al.,* 2012). RPL37, RPL37a, RPL38, RPL39, RPP0b, RPP0c, RPS21, RPS27, RPS30, RPS33, glycyl-tRNA synthetase, and recycling factor (RF) were not found (Additional file 5, Table S4).

***Pre-initiation complex:*** We found the core transcriptional pre-initiation complex proteins in *E. gracilis* (Additional file 5, Table S4). Proteins found include the general transcription factors, DNA helicases, RNA polymerases and activators and repressors. However, all or some members of TFIIA, B –TFIID, TFIID (BTAF1, BTF3, BTF3L4, EDF1, 6L, 7L, 11L), TFIIE, TFIIF, TFIIJ, TFIIK, RNAP, and the activators and repressors (CBP-1 and CTD kinases) (Maree and Patterson 2014; Blackwell and Walker 2006; Samorodnitsky and Pugh 2010; Yin and Wang 2014; Poss *et al.,* 2013; Rhee and Pugh 2012; Afek and Lukatsky 2013; Lauberth *et al.,* 2013; and Murakami *et al.,* 2013) were not found. Further analysis will be required to properly delineate the transcriptional apparatus in *E. gracilis.*

***Kinetochores*:** To identify proteins that are relevant for mitosis (conventional and non-conventional kinetochores), we used KKTs and KKIPs reported in *T. brucei* (Akiyoshi and Gull 2014, D’Archivio and Wickstead 2016) and a selection of eukaryotic conventional kinetochores as a reference set (Lampert and Westerman 2011, Meraldi *et al.* 2006, Kitagawa and Heiter 2001) (Additional file 5, Table S4). Out of the 19 KKTs and 7 KKIPs present in *T. brucei*, we found three in the *E. gracilis* transcriptome (KKT10, KKT19, and KKIP7) with multiple copies for KKT10/19. The KKT proteins show significantly greater homology to KKT10 than KKT19. Protein families of members of the microtubule plus-end, KMN, and centromeric interface, and centromeric DNA were identified (Lampert and Westerman, 2011, Meraldi*et al.* 2006, Kitagawa and Heiter, 2001) (Figure S8). Three complexes, MIS12, CCAN, and the CENPC, were not found in our dataset. Other proteins found are those involved in microtubule attachment, regulatory and signalling checkpoints, suggesting that the majority of the *Euglena* kinetochore is highly divergent.

***mRNA metabolism:*** We analyzed the *E. gracilis* transcriptome for mRNA metabolism gene candidates: DHH1, SCD6, XRNA, XRNB, XRNC, XRND, NOT1, and UPF1. Using BLAST, PSI-BLAST and OrthoFinder searches of reference *T. brucei* mRNA metabolism genes, we found the entire mRNA metabolism proteins from the reference set with moderate sequence similarity and amino acid conservation (Additional file 5, Table S4).

***Exosomes:*** Exosome proteins were identified in the *E. gracilis* proteome using T. brucei reference sets. Exosome proteins found include: RRP41A, RRP45, EAP1-4, RRP4, RRP40, DIS3-L, RRP44, and RRP6. RRP41B and CSL4 were not found (Additional file 5, Table S4).

***Spliceosomes and related proteins:*** The reference protein sequences for the predicted spliceosomal and related proteins are as contained in Yu *et al.* (2011) and Preußer *et al.*(2012). All subfamilies of the spliceosomal and related proteins were found (Additional file 5, Table S4). Some specific proteins within subfamilies were identified, including SF3b10 (U2). Subfamilies that were found include: SNRNPs (core proteins, major U2 type snRNP, minor U2 type snRNP, Tri-snRNP), non-snRNP (SR, PRP19 complex proteins, catalytic step II and late acting proteins, EJC, others with known motifs, others without know motifs), hnRNP proteins associated with the spliceosomes, and additional splicing regulators.

***Editosomes and related proteins:*** We have predicted the repertoire of mRNA editing and editosome proteins in *E. gracilis* using *T. brucei* (Salavati, et al., 2012, Deschamps, *et al.,* 2011, and Goringer, 2012) and *A. thaliana* reference sets (Takenaka, 2014) (Additional file 5, Table S4). These data suggest that *E. gracilis* is incapable of mitochondrial RNA editing.

***RNAi pathway:*** We searched for all the members of the RNA-induced silencing complex (RISC) using eukaryotic reference sets (Owens and Malham, 2015; Dunoyer, *et al.,* 2015; Dang, *et al.,* 2011). Present are all the subfamilies of the Dicer and Drosha - RNase III proteins, Argonaute proteins (except Zwille), small ncRNAs (except ssRNA binding), and Eri-1 like nuclease. RdRP and sid-1-like weren’t found (Additional file 5, Table S4)

***Galf:*** We found the glycosylinositol phospholipid Gal*f* transferase (GIPL galf), galactofuranosyltransferase lpg1-like protein (LPG1L), beta-galactofuranosyl glycosyltransferase (b-GalF), and beta-galactofuranosyl transferase (b-GalFT) (Stoco, *et al.,*2012; Tefsen, *et al.,* 2012; Matsunaga, et al., 2015; Komachi, et al., 2013) (Additional file 5, Table S4)

***PPG:*** Multiple copies of phosphoglycan beta (PPG) were found: PPG1, PPG3, PPG4, and PPG5, except for PPG2 which was not found (Table 11) (Satheesh, et al., 2014; Rogers, 2012; Secundino, et al., 2010; Eggimann, et al., 2015) (Additional file 5, Table S4)

***Carbohydrate biosynthesis:*** Using BLAST searches and the CAZy database, we found three superfamilies of Carbohydrate Active Enzymes: Glycoside Hydrolase (GH), Glycosyltransferases (GTs), and distant relatives of the Glycosyltransferases (GT-related) (Yoshida, et al., 2016; O’Neill and Field, 2015; Takeda, et al., 2015; Grimma, et al., 2015; O’Neill, et al., 2015A, B; Lombard, et al., 2013; Kuhaudomlarp, et al). We found 32, 36 and 10 families that are associated with the GHs, GTs, and GT-related respectively, with corresponding enzymatic activities ranging from 5'-AMP-activated protein kinase beta-subunit to glycosyltransferase (Additional file 5, Table S4).

***Metabolism in* Euglena*:*** There is a rich literature on enzymes of central metabolism, documenting how central intermediary metabolism of *E. gracilis* shifts in response to changes in environmental conditions. The transcripome sequences nonetheless still provides several metabolic surprises that offer questions for future experimentation and/or are significant in a wider evolutionary context.

A modified TCA cycle and unconventional attachment of heme to mitochondrial *c*-type cytochromes have been known for several decades^^[[1]](#footnote-1)^^. A striking characteristic of *E. gracilis* energy metabolism is that, under anaerobic conditions, mitochondrial ATP production switches from the canonical oxidative phosphorylation of heterotrophic eukaryotes (including most trypanosomatids) to an unusual fermentation involving rhodoquinone-dependent reduction of fumarate to succinate and wax-ester synthesis (Hoffmeister et al. 2005; 2004). Although rare, examples of rhodoquinone-dependent reduction of fumarate are known from other eukaryotes, but to our knowledge wax-ester fermentation is unique to *E. gracilis* and has been considered a potential biofuel. For instance, NADPH used to support the wax-ester synthesis is in part derived from oxidative decarboxylation of pyruvate to acetyl-CoA catalysed by pyruvate:NADP oxidoreductase (PNO). This enzyme substitutes the conventional pyruvate dehydrogenase enzyme in anaerobic conditions and it shows architectural and functional similarities to pyruvate:ferredoxin oxidoreductase (PFO) used by a variety of eukaryotes (Muller et al. 2012).

With unusual metabolic flexibility evident one might anticipate that, similar to a variety of other algae, notably *Chlamydomonas reinhardtii* (Catalanotti et al. 2013; Clowez et al. 2015), several other chlorophyte green algae (Ginger et al. 2010) *Cyanophora paradoxa* (Price et al. 2012) and possibly the centric diatom *Thalassiosira pseudonana* (Ginger et al. 2010), the *E. gracilis* genome would contain evidence of additional anaerobic traits deployable in response to appropriate environmental cues. However, *Euglena* appears to rely on a restricted, if highly unusual, set of pathways for anaerobic ATP production. In contrast to obligate anaerobic eukaryotes and algae (*C. reinhardtii*) or amoebae (*Acanthamoeba castellanii* and *N. gruberi*), in *Euglena* there appears to be capacity for neither acetate production with concomitant ATP production through substrate-level phosphorylation, nor for respiration via exogenous electron acceptors other than O_2_. Consistent with the literature (Tomita et al. 2016), lactate and succinate are predicted to be the only major end-products of anaerobic catabolism. A surprise offered by the transcriptome annotation is the presence of pyruvate:formate lyase (PFL) and its activating enzyme. This is the sole addition to the anaerobic repertoire of *E. gracilis*, except for the noted presence of a gene encoding flavodiiron protein, which is in some anaerobic protists important in the defense against reactive oxygen species. PFL seems to also become a common trait for chlorophyte green algae and protists such as *E. gracilis* which possess chloroplasts of green algal origin (Ginger et al. 2010). PFL, like pyruvate dehydrogenase, PNO, and PFO, provides another route for conversion of pyruvate to acetyl-CoA and is utilised by *Chlamydomonas* in both chloroplast and mitochondrion under anaerobic conditions (Atteia *et al.* 2006). The function and localization of PFL in *Euglena* is not known.

In summary, the paucity of anaerobic enzymes in *E. gracilis*, which has long been known for its adaptability to hypoxia and anoxia, stands in contrast to some other metabolically flexible eukaryotes, including its distant heterolobosean relation, the heterotrophic amoeboflagellate *N. gruberi*. It also contrasts with the diverse signaling networks in which signaling domains, such as the adenylate cyclase domains, are linked to PAS, BLUF or other domains associated with sensory perception that are seen in both *Euglena* and *Naegleria* and emphasize their adaptability towards variable environmental conditions.

Many pathways for amino acid biosynthesis absent from parasitic trypanosomatids and phagotrophic *B. saltans* and *N. gruberi* are present in *E. gracilis* (Additional file 5, Table S4). The presence of a single predicted polypeptide of 4121 amino acids encoding all the necessary subunits of a multifunctional fatty acid synthase (EG_transcript_9 and *cf* humans and yeast where the type I FAS has two sub-units) correlates well with early isolations of a cytosolic multi-functional fatty acid synthase from *Euglena* (Walker et al. 1981 inter alia). Yet, the presence of this enormous FAS contrasts with the trypanosomatids where elongases are unusually used for *de novo* fatty acid biosynthesis (Lee et al. 2006). *N. gruberi* is also both capable of *de novo* fatty acid biosynthesis but lacks a type I FAS, yet contains multiple elongases (Fritz-Laylin et al 2010), suggesting the pathway currently restricted to trypanosomatids may have a wider phylogenomic distribution. The type I FAS in *Euglena* indicates the presence of a more canonical textbook-style fatty acid biosynthetic pathway in an evolutionary divergent eukaryote.

The other major class of lipids required by most, if not all, eukaryotes are isoprenoids. Like other phototrophs, a methyl-d-erythritol pathway, presumably plastidic, is present, but a single HMG-CoA reductase is found in *Euglena* of the same length as the trypanosomatid enzyme. This lacks the N-terminal TMDs that characterise animal, yeast, and plant isoforms and confer endoplasmic reticulum localisation with the catalytic site facing the cytosol. Experimental descriptions of HMG-CoA reductases lacking N-terminal membrane spanning regions are restricted to the trypanosomatids and their similarity to the *E. gracilis* reductase raises the possibility that it has, like in the trypanosomatids, mitochondrial localization.

Irrespective of whether *E. gracilis* is kept under aerobic or anaerobic conditions, dihydroorotate dehydrogenase (DHOD) from the pyrimidine biosynthetic pathway is linked to respiratory chain activity, since in contrast to the trypanosomatids a fumarate-dependent DHOD is absent; a single gene predicted to encode the mitochondrial variant of DHOD is present. Predicted absence of a cytosolic DHOD correlates with the difference in fumarase isoforms present in trypanosomatids and *Euglena*: in the former, cytosolic and mitochondrial isoforms of type II fumarase are present, but in *E. gracilis* only one gene encodes type II fumarase. Fumarase activity in trypanosomatids is believed to be necessary for pyrimidine biosynthesis (providing fumarate as an essential electron sink; Coustou et al. 2006) and its activity may also be co-ordinated with the unusual soluble NADH-dependent fumarate reductase activity present in trypanosomatids. A single soluble candidate NADH-dependent fumarate reductase is also predicted for *E. gracilis*. The function (and localisation) of this enzyme in *E. gracilis*, if indeed it is not involved in rhodoquinone-dependent reduction of fumarate to succinate, is again enigmatic but it exists as single domain protein, in contrast to the large multi-domain mitochondrial and glycosomal fumarate reductases conserved in trypanosomatids and free-living kinetoplastid *B. saltans* (Coustou et al. 2005). Despite their many similarities, such differences again serve to highlight the very different evolutionary trajectories taken by the ancestors of heterotrophic kinetoplastids and the secondarily photosynthetic *E. gracilis*.

Finally, the shikimate pathway is present in *Euglena*, but again the genome annotation suggests a variation in pathway organisation not noted previously in eukaryotes: in contrast the pentafunctional form seen in fungi, ciliates, *Toxoplasma*, and *Phytophthora* or the individual proteins that each catalyse one reaction of shikimic acid biosynthesis which are found in other algae and plants (Richards et al. 2006), *E. gracilis* is predicted to encode a multifunctional protein with four of the five activities, and then a discrete protein catalyses the shikimate dehydrogenase reaction.

1. Like in the kinetoplastids there is no trace of the heme lyase or Ccm systems used to mature mitochondrial cytochromes *c* and *c*_1_ in *E. gracilis*. However, the hallmark components of *c*-type cytochrome maturation system II for heme attachment to chloroplast cytochromes are present (CCS1 ortholog, EG_transcript_3826; CCSA ortholog, EG_transcript_10248) . [↑](#footnote-ref-1)
